# One-Pot Synthesis of 3-Functionalized 4-Hydroxycoumarin under Catalyst-Free Conditions

**DOI:** 10.3390/molecules23010235

**Published:** 2018-01-22

**Authors:** Yang Gao, Guo-Ning Zhang, Juxian Wang, Xiaoguang Bai, Yiliang Li, Yucheng Wang

**Affiliations:** 1 Department of Pharmacy, Xuanwu Hospital Capital Medical University, Beijing 100053, China; gaoyang19820424@163.com; 2 Institute of Medicinal Biotechnology, Chinese Academy of Medical Science and Peking Union Medical College, Beijing 100050, China; raisunny2006@163.com (G.-N.Z.); imbjxwang@163.com (J.W.); baixiaoguang130@126.com (X.B.); 3 Tianjin Key Laboratory of Radiation Medicine and Molecular Nuclear Medicine, Institute of Radiation Medicine, Peking Union Medical College & Chinese Academy of Medical Sciences, Tianjin 300192, China

**Keywords:** 3-functionalized 4-hydroxycoumarin, multi-component domino reaction, catalyst-free, group-assisted purification process

## Abstract

A concise and efficient one-pot synthesis of 3-functionalized 4-hydroxycoumarin derivatives via a three-component domino reaction of 4-hydroxycoumarin, phenylglyoxal and 3-arylaminocyclopent-2-enone or 4-arylaminofuran-2(5*H*)-one under catalyst-free and microwave irradiation conditions is described. This synthesis involves a group-assisted purification process, which avoids traditional recrystallization and chromatographic purification methods.

## 1. Introduction

**Figure 1:**
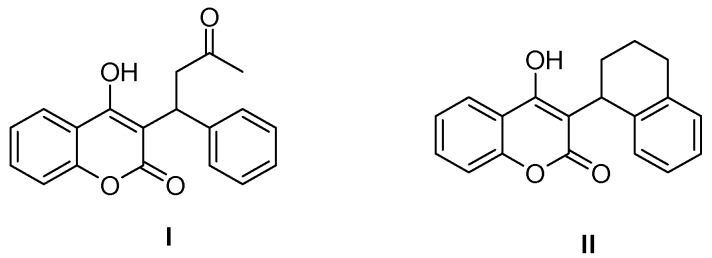
Biologically active coumarin derivatives.

Heterocyclic compounds are important because of their presence in a broad range of natural products and synthetic organic molecules with various biological activities [1,2]. Coumarin scaffolds are commonly found in diverse natural products, biologically active compounds and pharmaceuticals [3,4]. Among the various coumarin derivatives, substituted 4-hydroxycoumarin derivatives are of much importance because they exist in many natural products and exhibit a wide range of biological activities such as anti-HIV [5], anticancer [6], anti-coagulant [7] and antioxidant [8] activities. Warfarin I and coumatetralyl II are used for pesticides, specifically as a rodenticide and anticoagulant [9]. (Figure 1)

The development of a simple and eco-friendly protocol for the construction of heterocycles libraries of medical motifs is an attractive area of research in both academia and the pharmaceutical industry. Multicomponent reactions (MCRs) are promising and powerful tools in organic, combinatorial, and medicinal chemistry, because of their atom economy, complexity and diversity of products, multiple bond formation efficiency, and environmental friendliness [10]. These features make MCRs suitable for the easy construction of complex heterocyclic scaffolds from readily available starting materials [11]. In the past decade, some MCRs have been used for the construction of 4-hydroxycoumarin derivatives [12,13,14,15,16].

Microwave irradiation has been increasingly used in organic synthesis in recent years. Compared with traditional methods, this method has the advantages of higher yields, shorter reaction time, mild reaction conditions and environmentally friendliness. To date, a large number of organic reactions can be carried out under microwave irradiation conditions [17,18,19,20].

The development of environmentally friendly synthetic methods is a challenge in modern organic synthesis. The need to reduce the amount of toxic waste and byproducts arising from chemical processes has resulted in an increasing emphasis on the use of less-toxic and environmentally compatible materials in the design of new synthetic methods. Traditional purification methods such as recrystallization and column chromatography have problems in terms of consumption of organic solvents and energy, waste generation, and pollution. The concept of group-assisted purification (GAP) techniques, which avoid traditional crystallization and chromatographic purification methods and reduce waste generation from silica and solvents, particularly toxic solvents, was first developed by Li’s group in the design of asymmetric synthesis of new imine reagents [21,22]. To date, GAP chemistry has been used in many asymmetric reactions [23,24,25] and MCRs [26,27,28]. As part of our current studies on the development of environmentally friendly routes to heterocyclic compounds [29,30,31,32], we now report an efficient and clean synthesis of 3-functionalized 4-hydroxycoumarin derivatives under catalyst-free conditions.

## 2. Results and Discussion

We initially evaluated the three-component reaction of a 1:1:1 mixture of 4-hydroxycoumarin (**1**), phenylglyoxal monohydrate (**2a**) and 3-(*p*-tolylamino)cyclopent-2-enone (**3a**) for the optimization of the reaction conditions. The results are summarized in Table 1. The desired product **4a** was obtained in 89% yield when the reaction was carried out in ethanol at 100 °C for 30 min. under catalyst-free and microwave irradiation conditions (Table 1, entry 1). Various solvents were then evaluated to determine the impact of the solvent on the yield. Of all the solvents tested, i.e., anhydrous ethanol, water, DMF, acetonitrile, and a mixture of anhydrous ethanol-water (1:1 and 3:1, *v*/*v*), ethanol gave the best result (Table 1, entries 1–6). To improve the yield, several catalysts were evaluated: sodium hydrate, diethyl amine, *p*-toluenesulfonic acid (*p*-TSA), benzoic acid and *L*-proline (Table 1, entries 7–11). The results revealed that none of the catalysts could catalyze this reaction. The reaction was then conducted at different temperatures, such as 80, 90, 100 and 110 °C, to determine the optimum temperature for this transformation. All of these experiments were conducted in ethanol under catalyst-free and microwave irradiation conditions, and the desired product **4a** was obtained in yields of 69%, 76%, 89% and 87%, respectively (Table 1, entries 1 and 12–14). Finally, the reaction was performed at different reaction times to determine the optimum reaction time. The results showed that the best reaction time was 30 min (Table 1, entries 1 and 15–17). When the reaction was carried out in ethanol at refluxing temperature for 4 h in the absence of microwave, the desired product was obtained in 60% yield (Table 1, entry 18). These indicate that the microwave irradiation can improve the yield and shorten the reaction times. Accordingly, the best temperature for this transformation was 100 °C. On the basis of all of these experiments, the optimum reaction conditions were identified as ethanol at 100 °C for 30 min. under catalyst-free and microwave irradiation conditions.

With optimal conditions in hand, various substituted phenylglyoxal monohydrate (**2**) and 3-arylaminocyclopent-2-enone (**3**) were explored to investigate the generality of this three- component reaction for the synthesis of 3-functionalized 4-hydroxycoumarin derivatives (**4**). The results are tabulated in Table 2. The reaction seemed to be tolerant of substitution of the phenylglyoxal and 3-arylaminocyclopent-2-enone with either electron-withdrawing or electron-donating groups. Overall, yields in the range of 70%–95% were obtained.

To our delight, under optimal conditions, further experiments showed that when the 3-arylaminocyclopent-2-enone (**3**) was replaced by 4-arylaminofuran-2(5*H*)-one (**5**), the corresponding 3-functionalized 4-hydroxycoumarin derivatives (**6**) were obtained in good yields (Table 3).

It is important that this synthesis followed the GAP chemistry (group-assisted-purification chemistry) process, which can avoid traditional recrystallization or column chromatographic purification methods. Pure products were obtained simple by filtration and washing of the solid with a little cold ethanol.

**Figure 2:**
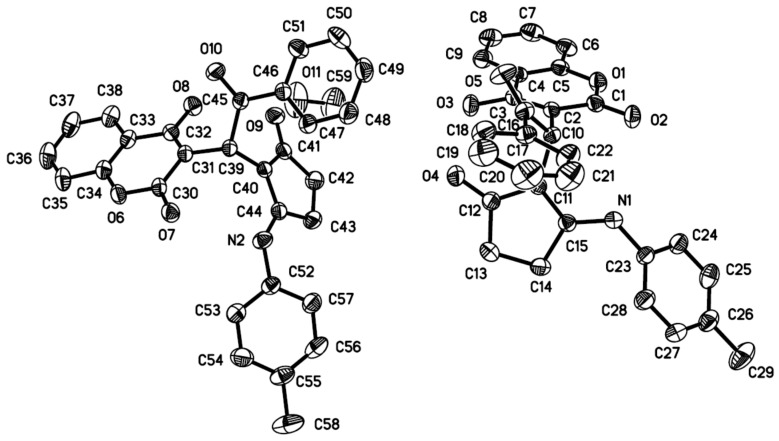
Crystal structure of compound **4a**.

The structures of compounds **4** and **6** were identified from their ^1^H NMR, and ^13^C NMR spectra, and by HRMS analysis. The structure of compound **4a** was further confirmed using single-crystal X-ray diffraction analysis (Figure 2).

**Figure 3:**
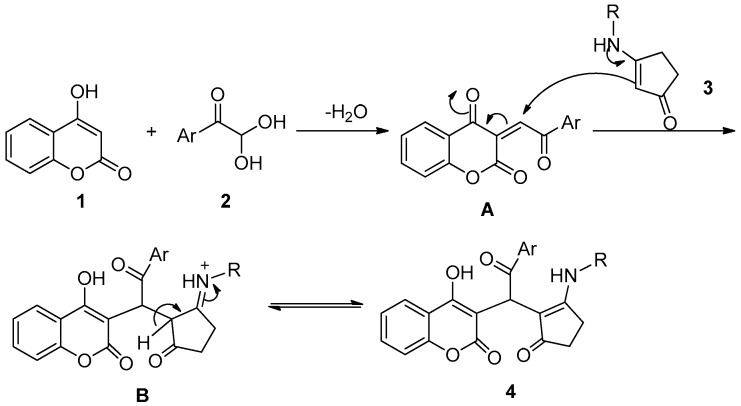
Proposed mechanism for the synthesis of compound **4**.

Although the detailed mechanism of this reaction remains to be fully clarified, the formation of compound **4** could be explained by the reaction sequence in Figure 3. First, a Knoevenagel condensation of 4-hydroxycoumarin **1** with phenylglyoxal **2** is proposed to give intermediate **A**. Michael addition of enaminone **3** to intermediate **A** then occurs to provide the intermediate **B**, which undergoes isomerization to form the desired product **4**.

## 3. Experimental

### 3.1. General

All reagents were commercial and used without further purification, unless otherwise indicated. Melting points were measured using an XT-4 micro melting point apparatus from Beijing Tech Instrument Co., Ltd., Beijing, China and were uncorrected. ^1^H NMR and ^13^C NMR spectra were recorded on Bruker Avance III HD-400 MHz spectrometer from Billerica, MA, USA in DMSO-*d*_6_ solution. *J* values are in hertz (Hz). Chemical shifts are expressed in *δ* downfield from internal tetramethylsilane (TMS). High-resolution mass spectra (HRMS) were obtained using Bruker MicrOTOF-Q II instrument from Billerica, MA, USA. X-ray crystal diffraction analysis was performed with a Bruker APEX-II CCD X-ray diffractometer from Billerica, MA, USA. Microwave irradiation was carried out with Initiator 2.5 Microwave Synthesizers from Biotage, Uppsala, Sweden. The reaction temperatures were measured by an infrared detector (external sensor type) during microwave heating.

### 3.2. General Procedure for the Synthesis of 3-Functionalized 4-Hydroxycoumarin Derivatives ***4*** and ***6***

4-Hydroxycoumarin (**1**) (0.5 mmol), substituted phenylglyoxal monohydrate (**2**) (0.5 mmol), and 3-arylaminocyclopent-2-enone (**3**) or 4-arylaminofuran-2(5*H*)-one (**5**) (0.5 mmol) were placed in a 10 mL Initiator reaction vial, followed by anhydrous ethanol (2 mL). The reaction vial was then sealed and prestirred for 15 s before being irradiated in the microwave (time, 30 min; temperature, 100 °C; absorption level, high; fixed hold time). The reaction mixture was then cooled to room temperature to give a precipitate, which was collected by Büchner filtration. The solid material was then washed with a little cold ethanol to afford the desired products **4** or **6**.

*4-Hydroxy-3-(2-oxo-1-(5-oxo-2-(p-tolylamino)cyclopent-1-en-1-yl)-2-phenylethyl)-2H-chromen-2-one* (**4a**). White solid, yield 86%, m.p. 134–135 °C. ^1^H NMR (400 MHz, DMSO-*d*_6_) *δ* 10.26 (s, 1H, OH), 7.78–7.71 (m, 3H, NH + ArH), 7.57–7.23 (m, 11H, ArH), 6.10 (s, 1H, CH), 3.03–2.96 (m, 1H, CH_2_), 2.71–2.64 (m, 1H, CH_2_), 2.47–2.42 (m, 2H, CH_2_), 2.32 (s, 3H, CH_3_). ^13^C NMR (100 MHz, DMSO-*d*_6_) *δ* 203.6, 196.4, 176.9, 164.8, 163.6, 152.0, 136.4, 135.5, 132.4, 132.2, 129.8, 128.0, 127.7, 124.0, 123.8, 123.7, 117.6, 115.9, 111.5, 105.0, 40.3, 31.7, 26.7, 20.4. HRMS (ESI) *m*/*z*: Calcd. for C_29_H_22_NO_5_ [M − H]^+^ 464.1498. Found: 464.1515.

*3-(1-(2-((4-Bromophenyl)amino)-5-oxocyclopent-1-en-1-yl)-2-oxo-2-(p-tolyl)ethyl)-4-hydroxy-2H-chromen-2-one* (**4b**). Brown solid, yield 90%, m.p. 139–140 °C. ^1^H NMR (400 MHz, DMSO-*d*_6_) *δ* 10.20 (s, 1H, OH), 7.76–7.67 (m, 3H, NH + ArH), 7.67–7.17 (m, 10H, ArH), 6.09 (s, 1H, CH), 3.09–3.02 (m, 1H, CH_2_), 2.76–2.70 (m, 1H, CH_2_), 2.48–2.38 (m, 2H, CH_2_), 2.25 (s, 3H, CH_3_). ^13^C NMR (100 MHz, DMSO-*d*_6_) *δ* 204.5, 195.6, 175.8, 164.3, 163.6, 160.3, 151.9, 142.7, 137.8, 133.6, 132.2, 128.7, 127.9, 125.4, 124.1, 123.8, 118.1, 117.5, 116.0, 112.5, 105.3, 99.5, 31.9, 26.7, 21.0. HRMS (ESI) *m*/*z*: Calcd. for C_29_H_23_BrNO_5_ [M + H]^+^ 544.0760. Found: 544.0754.

*3-(1-(2-((4-Chlorophenyl)amino)-5-oxocyclopent-1-en-1-yl)-2-oxo-2-(p-tolyl)ethyl)-4-hydroxy-2H-chromen-2-one* (**4c**). Brown solid, yield 79%, m.p. 142–143 °C. ^1^H NMR (400 MHz, DMSO-*d*_6_) *δ* 10.22 (s, 1H, OH), 7.77–7.67 (m, 3H, NH + ArH), 7.58–7.17 (m, 10H, ArH), 6.08 (s, 1H, CH), 3.09–3.01 (m, 1H, CH_2_), 2.75–2.69 (m, 1H, CH_2_), 2.47–2.41 (m, 2H, CH_2_), 2.25 (s, 3H, CH_3_). ^13^C NMR (100 MHz, DMSO-*d*_6_) *δ* 204.4, 195.7, 175.9, 164.4, 163.6, 151.9, 142.7, 137.4, 133.6, 132.2, 129.9, 129.3, 128.7, 127.9, 125.1, 124.1, 123.8, 117.5, 116.0, 112.5, 105.3, 99.5, 31.9, 30.6, 26.7, 21.0. HRMS (ESI) *m*/*z*: Calcd. for C_29_H_23_ClNO_5_ [M + H]^+^ 500.1265. Found: 500.1252.

*3-(1-(2-((4-Bromophenyl)amino)-5-oxocyclopent-1-en-1-yl)-2-(4-methoxyphenyl)-2-oxoethyl)-4-hydroxy-2H-chromen-2-one* (**4d**). Green solid, yield 85%, m.p. 222–223 °C. ^1^H NMR (400 MHz, DMSO-*d*_6_) *δ* 10.18 (s, 1H, OH), 7.77–7.74 (m, 3H, NH + ArH), 7.63–6.91 (m, 10H, ArH), 6.05 (s, 1H, CH), 3.74 (s, 3H, CH_3_O), 3.08–3.02 (m, 1H, CH_2_), 2.77–2.70 (m, 1H, CH_2_), 2.48–2.43 (m, 2H, CH_2_). ^13^C NMR (100 MHz, DMSO-*d*_6_) *δ* 204.5, 194.4, 175.7, 164.3, 163.6, 162.5, 152.0, 137.8, 132.2, 130.1, 128.7, 125.3, 124.1, 123.8, 118.0, 117.5, 116.0, 113.4, 112.7, 105.4, 55.3, 32.0, 26.7. HRMS (ESI) *m*/*z*: Calcd. for C_29_H_23_BrNO_6_ [M + H]^+^ 560.0709. Found: 560.0706.

*4-Hydroxy-3-(2-(4-methoxyphenyl)-2-oxo-1-(5-oxo-2-(p-tolylamino)cyclopent-1-en-1-yl)ethyl)-2H-chromen-2-one* (**4e**). Blue solid, yield 86%, m.p. 144–146 °C. ^1^H NMR (400 MHz, DMSO-*d*_6_) *δ* 10.22 (s, 1H, OH), 7.78–7.73 (m, 3H, NH + ArH), 7.58–6.90 (m, 10H, ArH), 6.05 (s, 1H, CH), 3.74 (s, 3H, CH_3_O), 3.01–2.95 (m, 1H, CH_2_), 2.71–2.64 (m, 1H, CH_2_), 2.47–2.37 (m, 2H, CH_2_), 2.41 (s, 3H, CH_3_). ^13^C NMR (100 MHz, DMSO-*d*_6_) *δ* 203.7, 194.5, 176.6, 164.7, 163.6, 162.5, 152.0, 135.7, 135.5, 132.1, 130.1, 129.8, 128.9, 124.0, 123.8, 123.7, 117.8, 115.9, 113.3, 111.8, 105.4, 55.3, 31.8, 26.7, 20.5. HRMS (ESI) *m*/*z*: Calcd. for C_30_H_26_NO_6_ [M + H]^+^ 496.1760. Found: 496.1757.

*4-Hydroxy-3-(2-(4-methoxyphenyl)-1-(2-((4-methoxyphenyl)amino)-5-oxocyclopent-1-en-1-yl)-2-oxoethyl)-2H-chromen-2-one* (**4f**). Brown solid, yield 81%, m.p. 138–139 °C. ^1^H NMR (400 MHz, DMSO-*d*_6_) *δ* 10.21 (s, 1H, OH), 7.77–7.72 (m, 3H, NH + ArH), 7.58–6.90 (m, 10H, ArH), 6.03 (s, 1H, CH), 3.77 (s, 3H, CH_3_O) 3.74 (s, 3H, OCH_3_), 2.94–2.88 (m, 1H, CH_2_), 2.65–2.58 (m, 1H, CH_2_), 2.45–2.36 (m, 2H, CH_2_). ^13^C NMR (100 MHz, DMSO-*d*_6_) *δ* 207.4, 194.5, 177.2, 164.9, 163.6, 162.5, 157.5, 152.0, 132.1, 130.9, 130.1, 128.8, 125.7, 124.0, 123.8, 117.9, 115.9, 114.5, 113.3, 111.3, 96.9, 55.3, 31.6, 26.7. HRMS (ESI) *m*/*z*: Calcd. for C_30_H_26_NO_7_ [M + H]^+^ 512.1709. Found 512.1693.

*3-(1-(2-((4-Bromophenyl)amino)-5-oxocyclopent-1-en-1-yl)-2-(4-chlorophenyl)-2-oxoethyl)-4-hydroxy-2H-chromen-2-one* (**4g**). White solid, yield 95%, m.p. 140–141 °C. ^1^H NMR (400 MHz, DMSO-*d*_6_) *δ* 10.24 (s, 1H, OH), 7.79–7.73 (m, 3H, NH + ArH), 7.64–7.25 (m, 10H, ArH), 6.10 (s, 1H, CH), 3.09–3.02 (m, 1H, CH_2_), 2.77–2.70 (m, 1H, CH_2_), 2.48–2.43 (m, 2H, CH_2_). ^13^C NMR (100 MHz, DMSO-*d*_6_) *δ* 204.8, 195.8, 176.5, 165.0, 164.1, 163.0, 152.5, 138.2, 137.8, 135.5, 132.7, 130.0, 128.8, 125.8, 124.3, 118.7, 117.8, 116.5, 113.9, 112.7, 105.3, 55.8, 40.8, 32.4, 27.2. HRMS (ESI) *m*/*z*: Calcd. for C_28_H_20_BrClNO_5_ [M + H]^+^ 564.0213. Found: 564.0203.

*3-(2-(4-Chlorophenyl)-2-oxo-1-(5-oxo-2-(p-tolylamino)cyclopent-1-en-1-yl)ethyl)-4-hydroxy-2H-chromen-2-one* (**4h**). Brown solid, yield 72%, m.p. 145–146 °C. ^1^H NMR (400 MHz, DMSO-*d*_6_) *δ* 10.27 (s, 1H, OH), 7.79–7.72 (m, 3H, NH + ArH), 7.58–7.24 (m, 10H, ArH), 6.10 (s, 1H, CH), 3.02–2.96 (m, 1H, CH_2_), 2.71–2.65 (m, 1H, CH_2_), 2.46–2.39 (m, 2H, CH_2_), 2.31 (s, 3H, CH_3_). ^13^C NMR (100 MHz, DMSO-*d*_6_) *δ* 206.2, 195.5, 177.0, 163.5, 152.0, 137.2, 135.6, 135.1, 132.2, 129.8, 129.6, 128.3, 124.0, 123.8, 123.6, 116.0, 111.4, 93.7, 40.3, 31.7, 26.5, 20.5. HRMS (ESI) *m*/*z*: Calcd. for C_29_H_23_ClNO_5_ [M + H]^+^ 500.1265. Found 500.1257.

*3-(2-(4-Chlorophenyl)-1-(2-((4-methoxyphenyl)amino)-5-oxocyclopent-1-en-1-yl)-2-oxoethyl)-4-hydroxy-2H-chromen-2-one* (**4i**). Brown solid, yield 83%, m.p. 140–141 °C. ^1^H NMR (400 MHz, DMSO-*d*_6_) *δ* 10.29 (s, 1H, OH), 7.79–7.72 (m, 3H, NH + ArH), 7.57–6.99 (m, 10H, ArH), 6.08 (s, 1H, CH), 3.77 (s, 3H, CH_3_O), 2.96–2.90 (m, 1H, CH_2_), 2.66–2.59 (m, 1H, CH_2_), 2.44–2.37 (m, 2H, CH_2_). ^13^C NMR (100 MHz, DMSO-*d*_6_) *δ* 203.1, 195.5, 177.5, 165.2, 163.5, 157.6, 152.0, 137.2, 135.1, 132.1, 130.7, 129.5, 128.3, 125.6, 123.9, 123.8, 117.8, 115.9, 114.5, 110.9, 104.6, 55.3, 40.3, 31.6, 26.6. HRMS (ESI) *m*/*z*: Calcd. for C_29_H_23_ClNO_5_ [M + H]^+^ 500.1265. Found 500.1257.

*3-(2-(4-Bromophenyl)-1-(2-((4-bromophenyl)amino)-5-oxocyclopent-1-en-1-yl)-2-oxoethyl)-4-hydroxy-2H-chromen-2-one* (**4j**). Blue solid, yield 70%, m.p. 159–160 °C. ^1^H NMR (400 MHz, DMSO-*d*_6_) *δ* 10.24 (s, 1H, OH), 7.76–7.68 (m, 3H, NH + ArH), 7.64–7.25 (m, 10H, ArH), 6.08 (s, 1H, CH), 3.09–3.02 (m, 1H, CH_2_), 2.77–2.71 (m, 1H, CH_2_), 2.47–2.38 (m, 2H, CH_2_). ^13^C NMR (100 MHz, DMSO-*d*_6_) *δ* 204.3, 195.5, 176.0, 164.6, 163.5, 152.0, 137.7, 135.4, 132.3, 132.2, 131.3, 129.7, 126.4, 125.3, 124.1,123.8, 118.2, 117.4, 116.0, 112.3, 104.7, 89.6, 40.3, 31.9, 26.7. HRMS (ESI) *m*/*z*: Calcd. for C_28_H_20_Br_2_NO_5_ [M + H]^+^ 607.9708. Found 607.9698.

*3-(2-(4-Bromophenyl)-2-oxo-1-(5-oxo-2-(p-tolylamino)cyclopent-1-en-1-yl)ethyl)-4-hydroxy-2H-chromen-2-one* (**4k**). Brown solid, yield 77%, m.p. 161–162 °C. ^1^H NMR (400 MHz, DMSO-*d*_6_) *δ* 10.27 (s, 1H, OH), 7.75–7.69 (m, 3H, NH + ArH), 7.63–7.24 (m, 10H, ArH), 6.09 (s, 1H, CH), 3.02–2.96 (m, 1H, CH_2_), 2.71–2.65 (m, 1H, CH_2_), 2.45–2.38 (m, 2H, CH_2_), 2.31 (s, 3H, CH_3_). ^13^C NMR (100 MHz, DMSO-*d*_6_) *δ* 203.5, 195.6, 177.0, 165.0, 163.5, 152.0, 135.6, 135.4, 132.2, 131.2, 139.8, 129.7, 126.3, 124.0,123.8, 123.6, 117.7, 116.0, 111.4, 89.7, 40.3, 31.7, 26.8, 20.5. HRMS (ESI) *m*/*z*: Calcd. for C_29_H_23_BrNO_5_ [M + H]^+^ 544.0760. Found 544.0762.

*3-(1-(4-((4-Bromophenyl)amino)-2-oxo-2,5-dihydrofuran-3-yl)-2-oxo-2-phenylethyl)-4-hydroxy-2H-chromen-2-one* (**6a**). Pink solid, yield 89%, m.p. 221–223 °C. ^1^H NMR (400 MHz, DMSO-*d*_6_) *δ* 9.51 (s, 1H, OH), 7.96–7.80 (m, 3H, NH + ArH), 7.62–7.07 (m, 10H, ArH), 5.94 (s, 1H, CH), 5.25 (d, *J* = 15.6Hz, 1H, CH_2_), 5.15 (d, *J* = 16.0 Hz, H, CH_2_). ^13^C NMR (400 MHz, DMSO-*d*_6_) *δ* 195.8, 174.7, 163.4, 162.8, 162.2, 152.0, 138.6, 132.6, 132.3, 128.3, 127.7, 124.2, 123.7, 122.0, 116.4, 116.3, 115.8, 104.3, 94.6, 66.6, 40.4. HRMS (ESI) *m*/*z*: Calcd. for C_27_H_19_BrNO_6_ [M + H]^+^ 532.0396. Found 532.0402.

*4-Hydroxy-3-(1-(4-((4-methoxyphenyl)amino)-2-oxo-2,5-dihydrofuran-3-yl)-2-oxo-2-phenylethyl)-2H-chromen-2-one* (**6b**). Brown solid, yield 84%, m.p. 116–118 °C. ^1^H NMR (400 MHz, DMSO-*d*_6_) *δ* 9.29 (s, 1H, OH), 7.93–7.79 (m, 3H, ArH + NH), 7.62–6.91 (m, 11H, ArH), 5.95 (s, 1H, CH), 5.08 (d, *J* = 16.0 Hz, 1H, CH_2_), 4.89 (d, *J* = 16.0 Hz, 1H, CH_2_), 3.73 (s, 3H, CH_3_O). ^13^C NMR (400 MHz, DMSO-*d*_6_) *δ* 196.0, 175.6, 163.7, 163.2, 162.6, 156.5, 151.9, 136.3, 132.4, 131.6, 128.2, 127.6, 124.1, 123.6, 123.2, 116.3, 114.9, 114.6, 104.6, 92.0, 66.5, 55.2, 40.2. HRMS (ESI) *m*/*z*: Calcd. for C_27_H_19_BrNO_6_ [M + H]^+^ 484.1396. Found 484.1402.

*3-(2-(4-Chlorophenyl)-2-oxo-1-(2-oxo-4-(p-tolylamino)-2,5-dihydrofuran-3-yl)ethyl)-4-hydroxy-2H-chromen-2-one* (**6c**). White solid, yield 92%, m.p. 219–220 °C. ^1^H NMR (400 MHz, DMSO-*d*_6_) *δ* 9.30 (s, 1H, OH), 7.97–7.80 (m, 3H, NH + ArH), 7.62–7.03 (m, 10H, ArH), 5.94 (s, 1H, CH), 5.16 (d, 1H, *J* = 15.6 Hz, CH_2_), 5.06 (d, *J* = 16.0 Hz, 1H, CH_2_), 2.25 (s, 3H, CH_3_). ^13^C NMR (400 MHz, DMSO-*d*_6_) *δ* 194.9, 174.8, 163.1, 162.8, 152.0, 137.2, 136.4, 135.1, 133.5, 132.4, 129.9, 129.4, 128.4, 124.1, 123.6, 120.6, 116.3, 104.0, 92.7, 66.5, 56.0, 40.4, 20.3, 18.5. HRMS (ESI) *m*/*z*: Calcd. for C_28_H_21_ClNO_6_ [M + H]^+^ 502.1057. Found 502.1068.

*3-(2-(4-Chlorophenyl)-1-(4-((4-chlorophenyl)amino)-2-oxo-2,5-dihydrofuran-3-yl)-2-oxoethyl)-4-hydroxy-2H-chromen-2-one* (**6d**). Pink solid, yield 70%, m.p. 208–210 °C. ^1^H NMR (400 MHz, DMSO-*d*_6_) *δ* 9.53 (s, 1H, OH), 7.96–7.79 (m, 3H, NH + ArH), 7.62–7.12 (m, 10H, ArH), 5.87 (s, 1H, CH), 5.21 (d, *J* = 16.0 Hz, 1H, CH_2_), 5.12 (d, *J* = 16.0 Hz, 1H, CH_2_). ^13^C NMR (400 MHz, DMSO-*d*_6_) *δ* 194.8, 174.3, 163.1, 162.1, 152.1, 138.1, 137.2, 135.1, 132.4, 129.4, 128.4, 127.7, 124.1, 123.6, 121.7, 116.4, 103.7, 94.2, 66.4, 40.2. HRMS (ESI) *m*/*z*: Calcd. for C_27_H_18_Cl_2_NO_6_ [M + H]^+^ 522.0521. Found 522.0523.

*3-(1-(4-((4-Bromophenyl)amino)-2-oxo-2,5-dihydrofuran-3-yl)-2-(4-chlorophenyl)-2-oxoethyl)-4-hydroxy-2H-chromen-2-one* (**6e**). Pink solid, yield 91%, m.p. 190–191 °C. ^1^H NMR (400 MHz, DMSO-*d*_6_) *δ* 9.46 (s, 1H, OH), 7.97–7.80 (m, 3H, NH + ArH), 7.62–7.08 (m, 10H, ArH), 5.91 (s, 1H, CH), 5.24 (d, *J* = 16.0 Hz, 1H, CH_2_), 5.14 (d, *J* = 16.0 Hz, 1H, CH_2_). ^13^C NMR (400 MHz, DMSO-*d*_6_) *δ* 195.2, 174.8, 163.8, 163.3, 162.6, 152.5, 139.0, 137.8, 135.5, 132.8, 132.6, 129.9, 128.9, 124.6, 124.1, 122.5, 116.8, 116.2, 104.3, 94.8, 67.0, 19.0. HRMS (ESI) *m*/*z*: Calcd. for C_27_H_18_BrClNO_6_ [M + H]^+^ 566.0006. Found 566.0026.

*3-(2-(4-Bromophenyl)-2-oxo-1-(2-oxo-4-(p-tolylamino)-2,5-dihydrofuran-3-yl)ethyl)-4-hydroxy-2H-chromen-2-one* (**6f**). Pink solid, yield 91%, m.p. 218–219 °C. ^1^H NMR (400 MHz, DMSO-*d*_6_) *δ* 9.31 (s, 1H, OH), 7.96–7.71 (m, 3H, NH + ArH), 7.65–7.02 (m, 10H, ArH), 5.91 (s, 1H, CH), 5.15 (d, *J* = 16.0 Hz, 1H, CH_2_), 5.05 (d, *J* = 16.0 Hz, 1H, CH_2_), 5.25 (s, 3H, CH_3_). ^13^C NMR (100 MHz, DMSO-*d*_6_) *δ* 195.2, 174.7, 163.1, 162.9, 152.0, 136.4, 135.5, 133.4, 132.4, 131.3, 129.9, 129.5, 126.3, 124.1, 123.6, 120.6, 116.4, 103.9, 92.7, 66.4, 20.3, 18.5. HRMS (ESI) *m*/*z*: Calcd. for C_28_H_21_BrNO_6_ [M + H]^+^ 546.0552. Found 546.0543.

*3-(2-(4-Bromophenyl)-1-(4-((4-methoxyphenyl)amino)-2-oxo-2,5-dihydrofuran-3-yl)-2-oxoethyl)-4-hydroxy-2H-chromen-2-one* (**6g**). White solid, yield 77%, m.p. 155–157 °C. ^1^H NMR (400 MHz, DMSO-*d*_6_) *δ* 9.27 (s, 1H, OH), 7.93–7.70 (m, 3H, NH + ArH), 7.73–6.90 (m, 10H, ArH), 5.86(s, 1H, CH), 5.05 (d, *J* = 15.6 Hz, 1H, CH_2_), 4.95 (d, *J* = 16.0 Hz, 1H, CH_2_), 3.73 (s, 3H, CH_3_). ^13^C NMR (100 MHz, DMSO-*d*_6_) *δ* 195.1, 175.2, 163.7, 163.1, 162.6, 156.5, 152.0, 135.5, 132.4, 131.6, 131.3, 129.5, 126.4, 124.1, 123.6, 123.2, 116.3, 114.6, 104.2, 91.7, 66.3, 55.2, 40.7. HRMS (ESI) *m*/*z*: Calcd. for C_28_H_21_BrNO_7_ [M + H]^+^ 562.0501. Found 562.0493.

*4-Hydroxy-3-(2-(4-methoxyphenyl)-1-(4-((4-methoxyphenyl)amino)-2-oxo-2,5-dihydrofuran-3-yl)-2-oxoethyl)-2H-chromen-2-one* (**6h**). White solid, yield 91%, m.p. 170–172 °C. ^1^H NMR (400 MHz, DMSO-*d*_6_) *δ* 9.28 (s, 1H, OH), 7.94–7.77 (m, 3H, NH + ArH), 7.63–6.91 (m, 10 H, ArH), 5.92(s, 1H, CH), 5.19 (d, *J* = 15.6 Hz, 1H, CH_2_), 4.97 (d, *J* = 15.6 Hz, 1H, CH_2_), 3.76 (s, 3H, CH_3_O), 3.73 (s, 3H, CH_3_O). ^13^C NMR (100 MHz, DMSO-*d*_6_) *δ* 194.3, 176.0, 163.7, 163.3, 162.6, 156.5, 151.9, 132.4, 131.6, 129.9, 128.7, 124.1, 123.6, 123.1, 116.3, 114.6, 113.5, 105.0, 92.4, 66.6, 55.2, 39.7. HRMS (ESI) *m*/*z*: Calcd. for C_29_H_24_NO_8_ [M + H]^+^ 514.1502. Found 514.1495.

*3-(1-(4-((4-Chlorophenyl)amino)-2-oxo-2,5-dihydrofuran-3-yl)-2-(4-methoxyphenyl)-2-oxoethyl)-4-hydroxy-2H-chromen-2-one* (**6i**). Brown solid, yield 72%, m.p. 206–207. ^1^H NMR (400 MHz, DMSO-*d*_6_) *δ* 9.48 (s, 1H, OH), 7.97–7.78 (m, 3H, NH + ArH), 7.61–6.94 (m, 10H, ArH), 5.93 (s, 1H, CH), 5.25 (d, *J* = 16.0 Hz, 1H, CH_2_), 5.14 (d, *J* = 16.0 Hz, 1H, CH_2_), 3.76 (s, 3H, CH_3_O). ^13^C NMR (100 MHz, DMSO-*d*_6_) *δ* 194.1, 174.9, 163.4, 162.6, 162.1, 152.0, 138.2, 132.4, 129.9, 129.4, 128.7, 127.6, 124.1, 123.6, 121.5, 116.3, 113.5, 104.4, 94.8, 66.6, 55.3, 40.0. HRMS (ESI) *m*/*z*: Calcd. for C_28_H_21_ClNO_7_ [M + H]^+^ 518.1007. Found 518.1018. 

## 4. Conclusions

In summary, we have developed a novel, highly efficient, catalyst-free, green protocol for the one-pot three-component synthesis of 3-functionalized 4-hydroxycoumarin derivatives. This protocol has the advantages of mild reaction conditions, high yields, convenient operation, and environmental friendliness.

## Figures and Tables

**Table 1:**
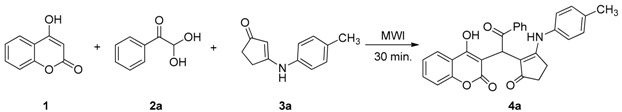
Optimization of the reaction conditions.

Entry	Catalyst (mol %)	Solvent (*v*/*v*)	Temperature (°C)	Time (min)	Yield (%) ^a^
1	No	EtOH	100	30	89
2	No	H_2_O	100	30	36
3	No	DMF	100	30	70
4	No	CH_3_CN	100	30	80
5	No	EtOH-H_2_O (1:1)	100	30	54
6	No	EtOH:H_2_O (3:1)	100	30	72
7	NaOH (20)	EtOH	100	30	33
8	Et_2_NH (20)	EtOH	100	30	50
9	*p*-TSA (20)	EtOH	100	30	80
10	Benzoic Acid (20)	EtOH	100	30	51
11	*L*-Proline (20)	EtOH	100	30	81
12	No	EtOH	80	30	69
13	No	EtOH	90	30	76
14	No	EtOH	110	30	87
15	No	EtOH	100	10	57
16	No	EtOH	100	20	65
17	No	EtOH	100	40	86
18	No	EtOH	Reflux (absence of microwave)	240	60

^a^ Yield was determined by HPLC-MS.

**Table 2:**
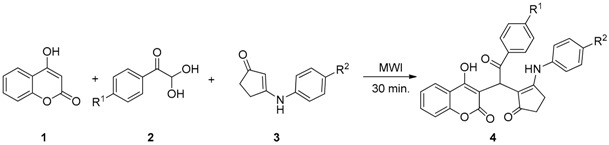
Synthesis of 3-functionalized 4-hydroxycoumarin derivatives **4**.

Entry	R^1^	R^2^	Product	Isolated Yield (%)
1	H	CH_3_	**4a**	86
2	CH_3_	Br	**4b**	90
3	CH_3_	Cl	**4c**	79
4	CH_3_O	Br	**4d**	85
5	CH_3_O	CH_3_	**4e**	86
6	CH_3_O	CH_3_O	**4f**	81
7	Cl	Br	**4g**	95
8	Cl	CH_3_	**4h**	72
9	Cl	CH_3_O	**4i**	83
10	Br	Br	**4j**	70
11	Br	CH_3_	**4k**	77

**Table 3:**
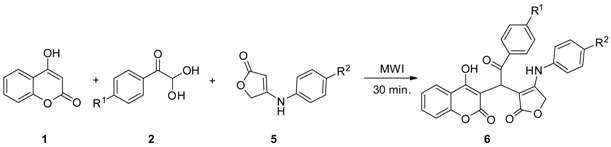
Synthesis of 3-functionalized 4-hydroxycoumarin derivatives **6**.

Entry	R^1^	R^2^	Product	Isolated Yield (%)
1	H	Br	**6a**	89
2	H	CH_3_O	**6b**	84
3	Cl	CH_3_	**6c**	92
4	Cl	Cl	**6d**	70
5	Cl	Br	**6e**	91
6	Br	CH_3_	**6f**	91
7	Br	CH_3_O	**6g**	77
8	CH_3_O	CH_3_O	**6h**	91
9	CH_3_O	Cl	**6i**	72

## Supplementary Materials

Supplementary materials are available online. ^1^H NMR and ^13^C NMR spectrum of compounds **4a**–**4k** and **6a**–**6i**.
