# Supplementary material for: One-Pot Synthesis of 3-Functionalized 4-Hydroxycoumarin under Catalyst-Free Conditions
Source: Molecules. 2018 Jan 22;23(1):235. doi: 10.3390/molecules23010235 (PMC6017609; doi:10.3390/molecules23010235)

(Supplementary Materials)

One-Pot Syntesis of 3-Functionalized 4-Hydroxy- coumarin under Catalyst-Free Conditions

Yang Gao [1], Guoning Zhang[2], Juxian Wang[2], Xiaoguang Bai[2], Yiliang Li[3,]* and Yucheng Wang [2,]*

^{1}H NMR of compound **4a**

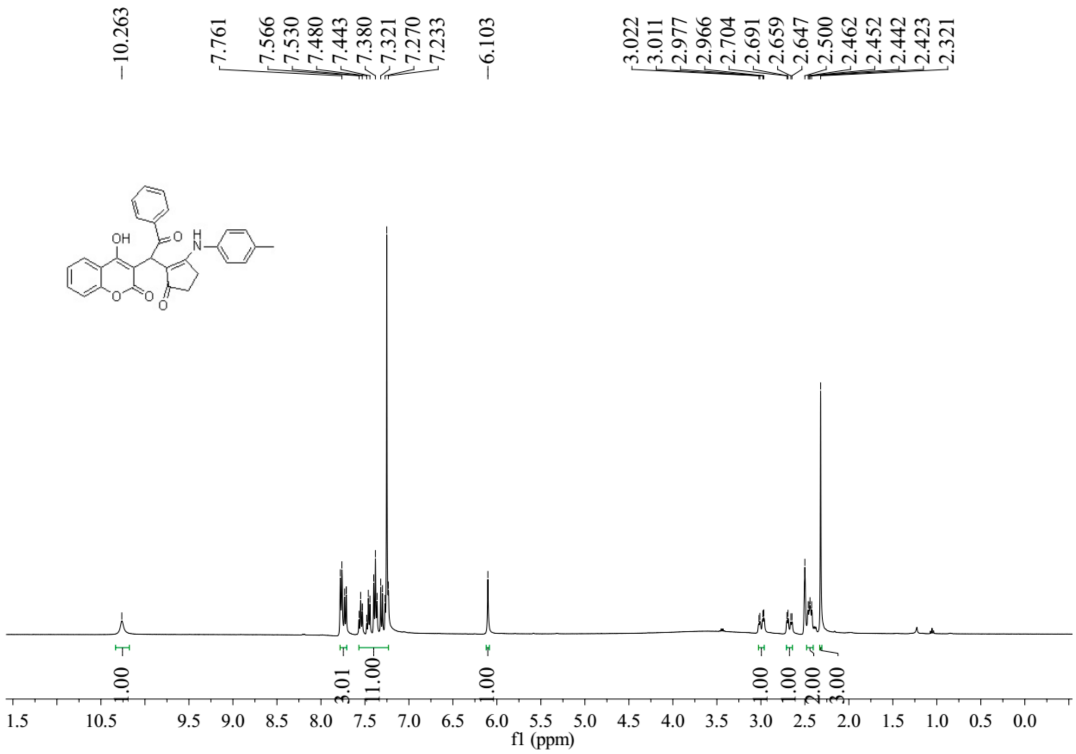

^{13}C NMR of compound **4a**

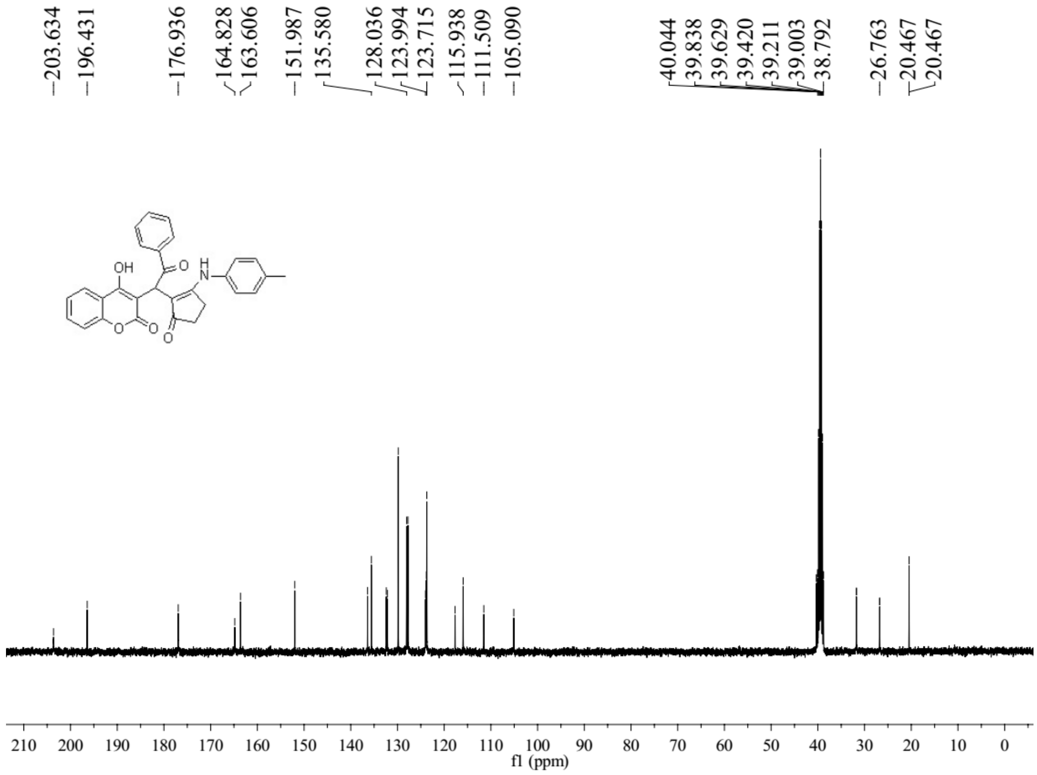

^{1}H NMR of compound **4b**

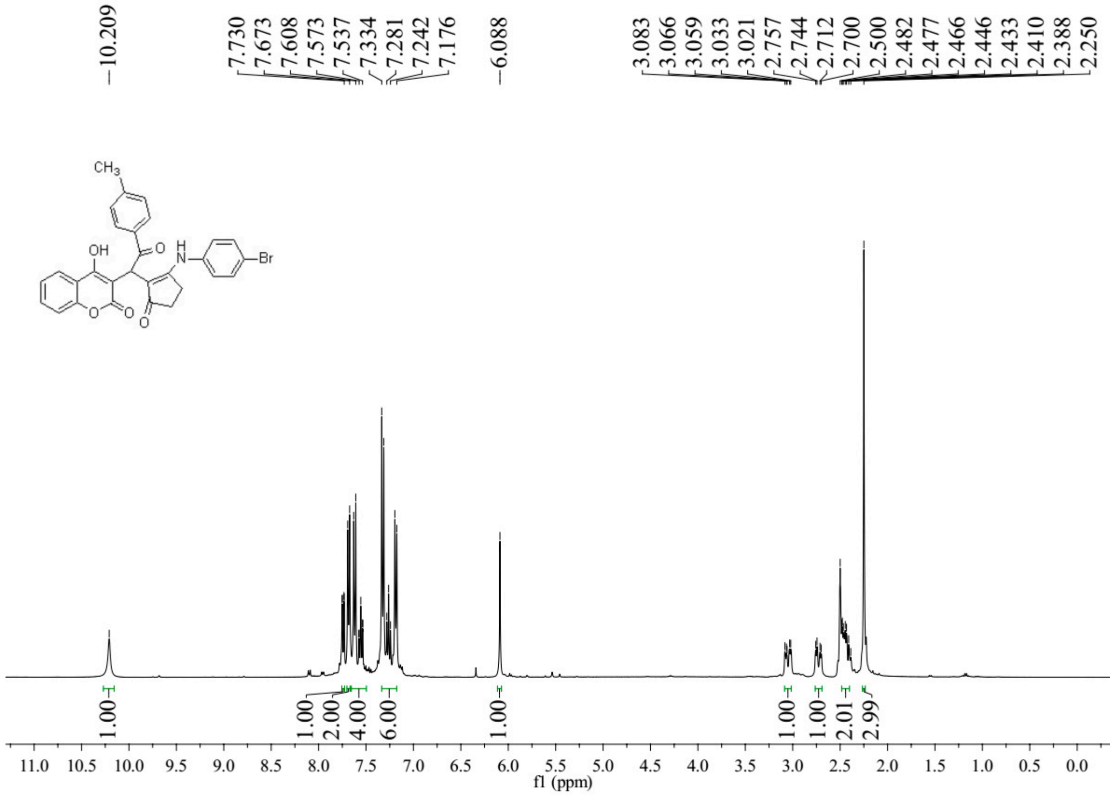

^{13}C NMR of compound **4b**

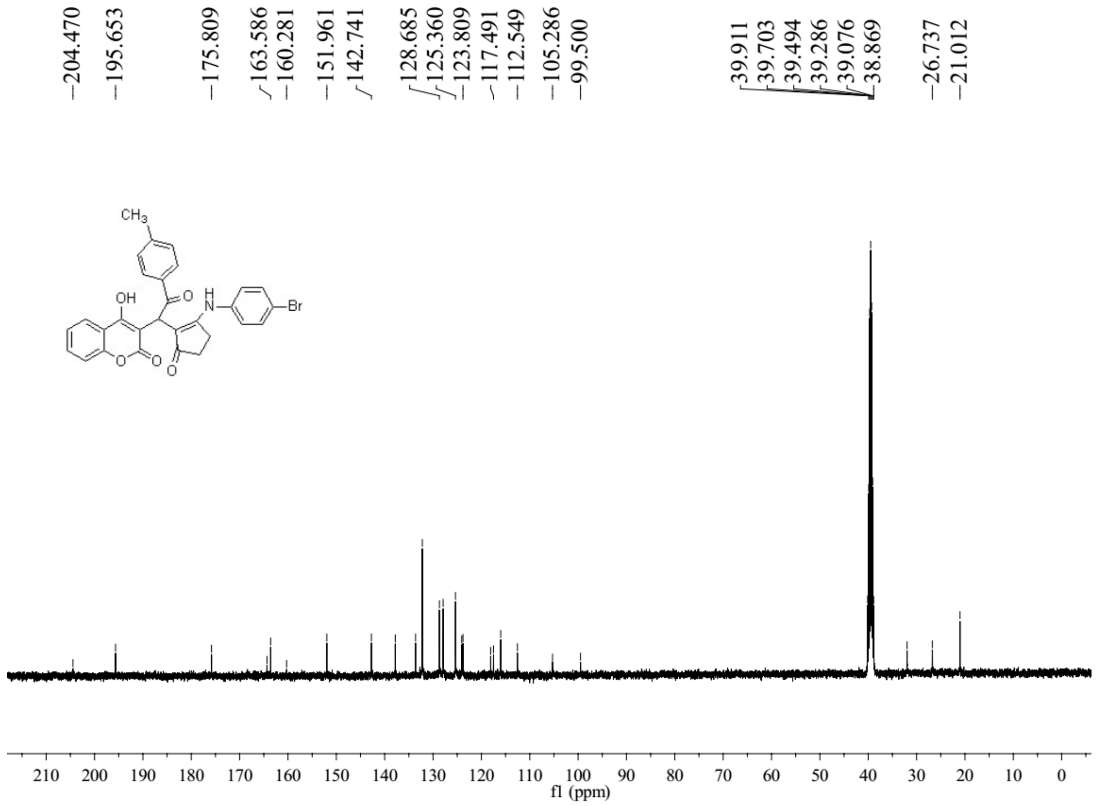

^{1}H NMR of compound **4c**

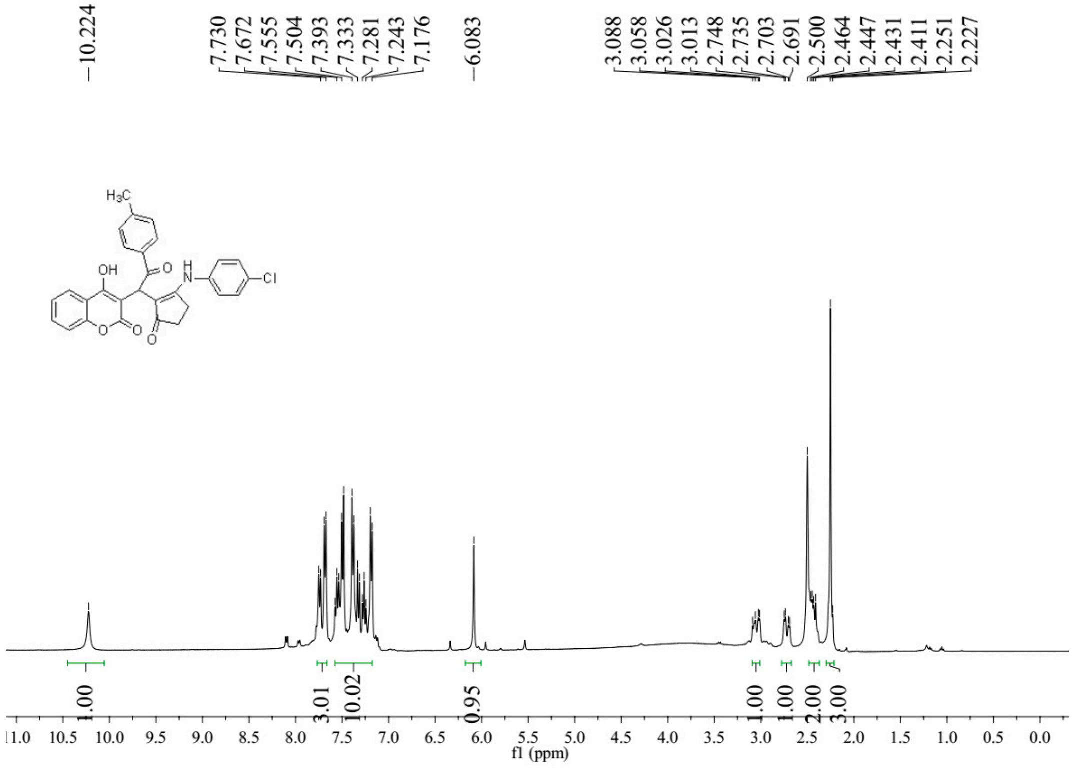

^{13}C NMR of compound **4c**

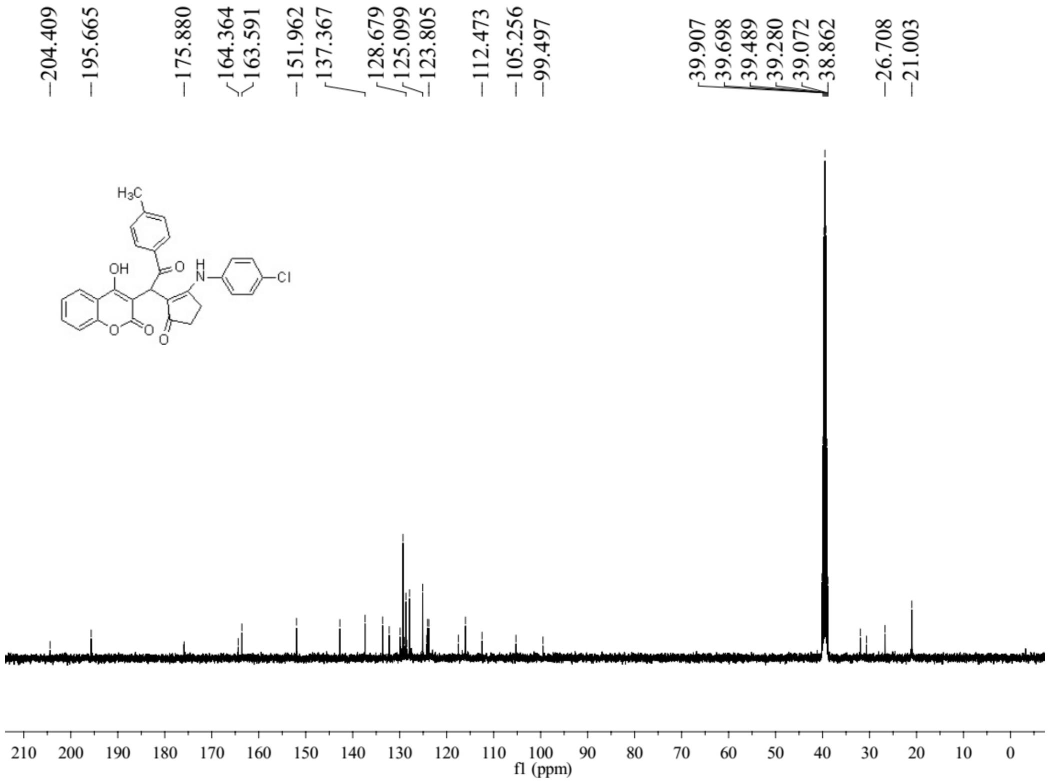

^{1}H NMR of compound **4d**

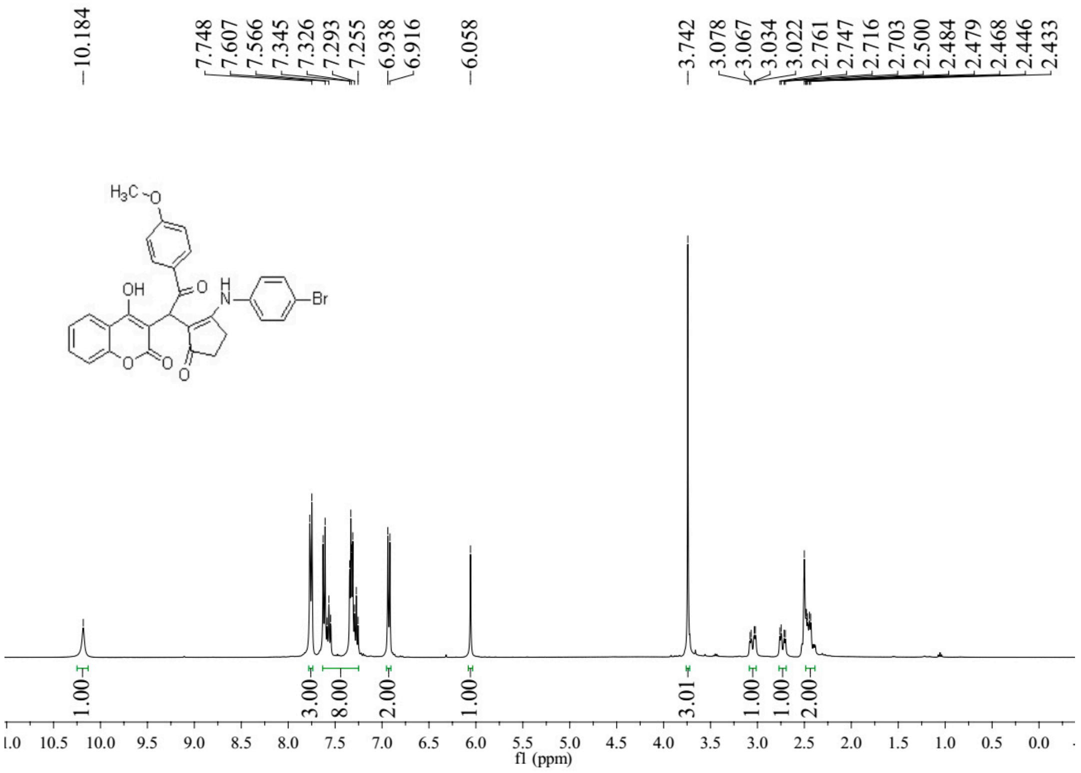

^{13}C NMR of compound **4d**

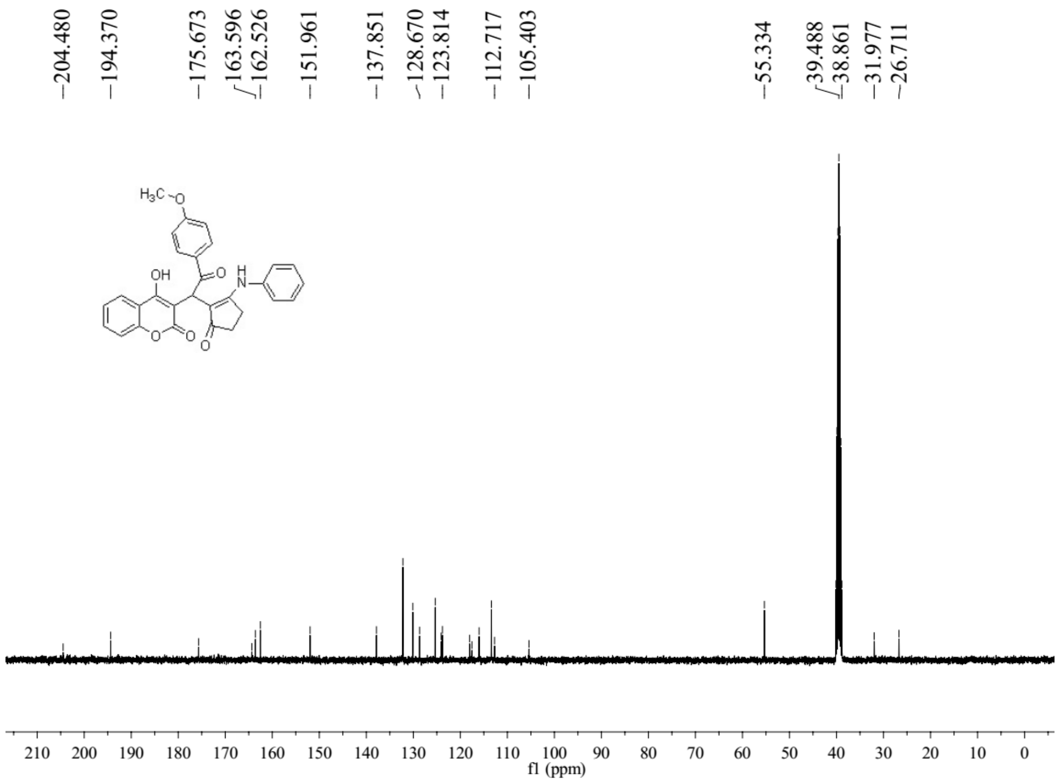

^{1}H NMR of compound **4e**

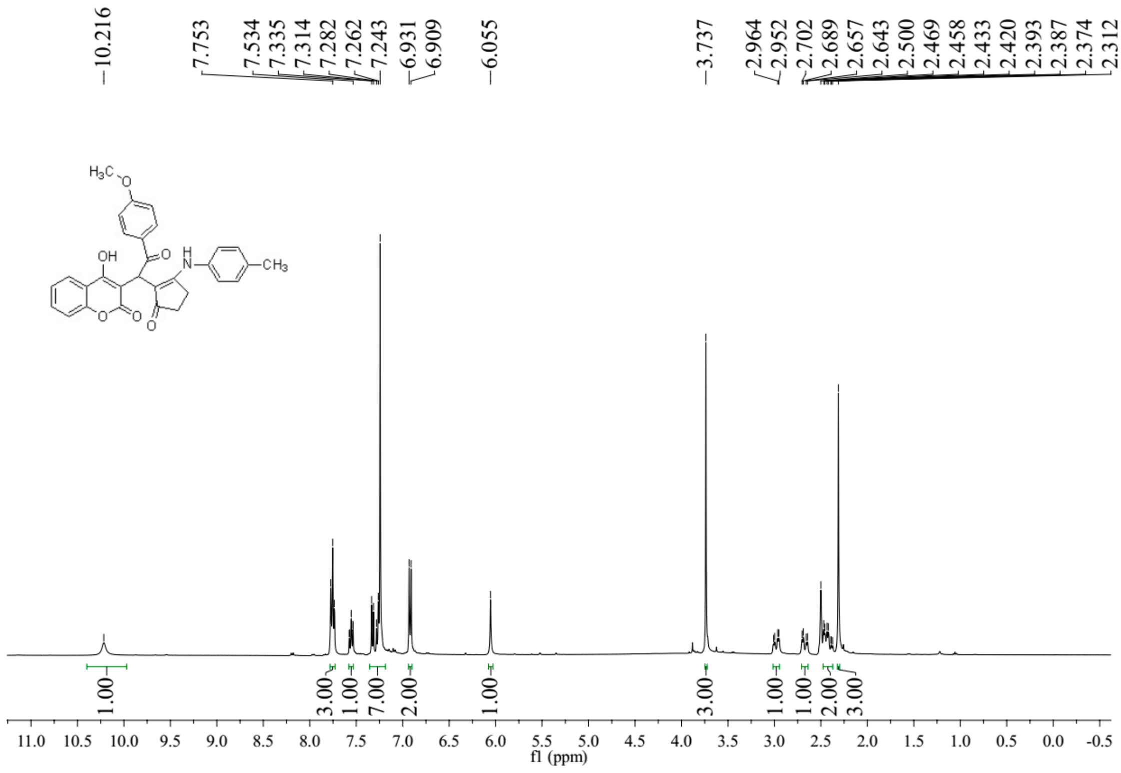

^{13}C NMR of compound **4e**

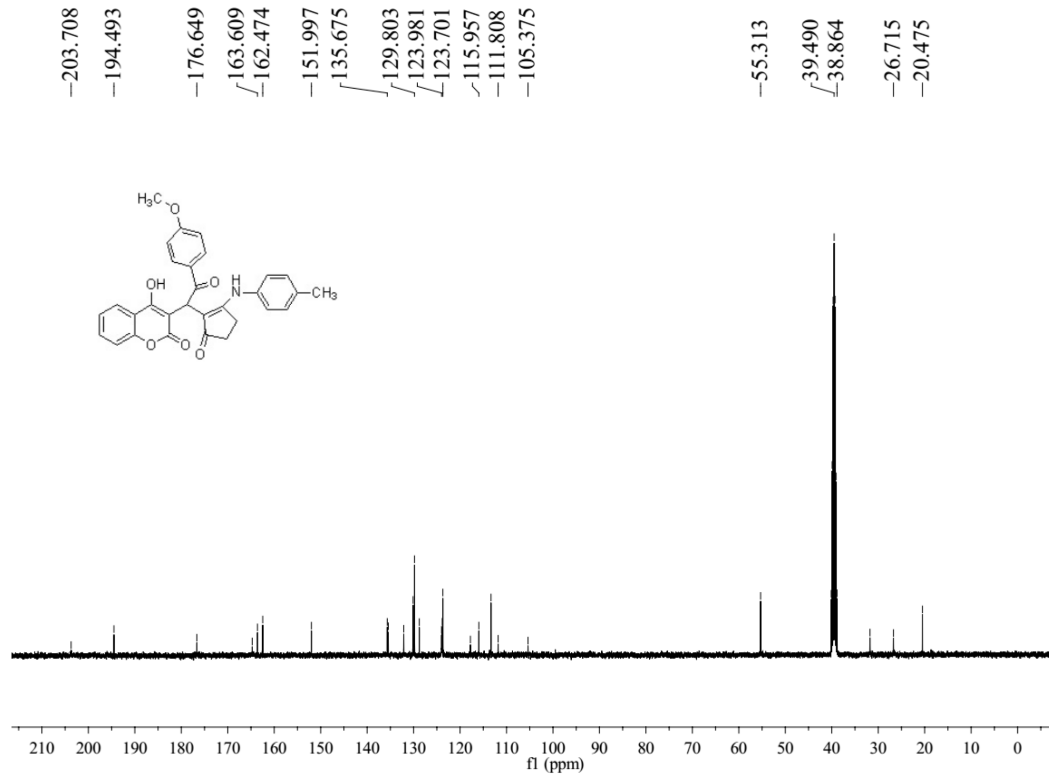

^{1}H NMR of compound **4f**

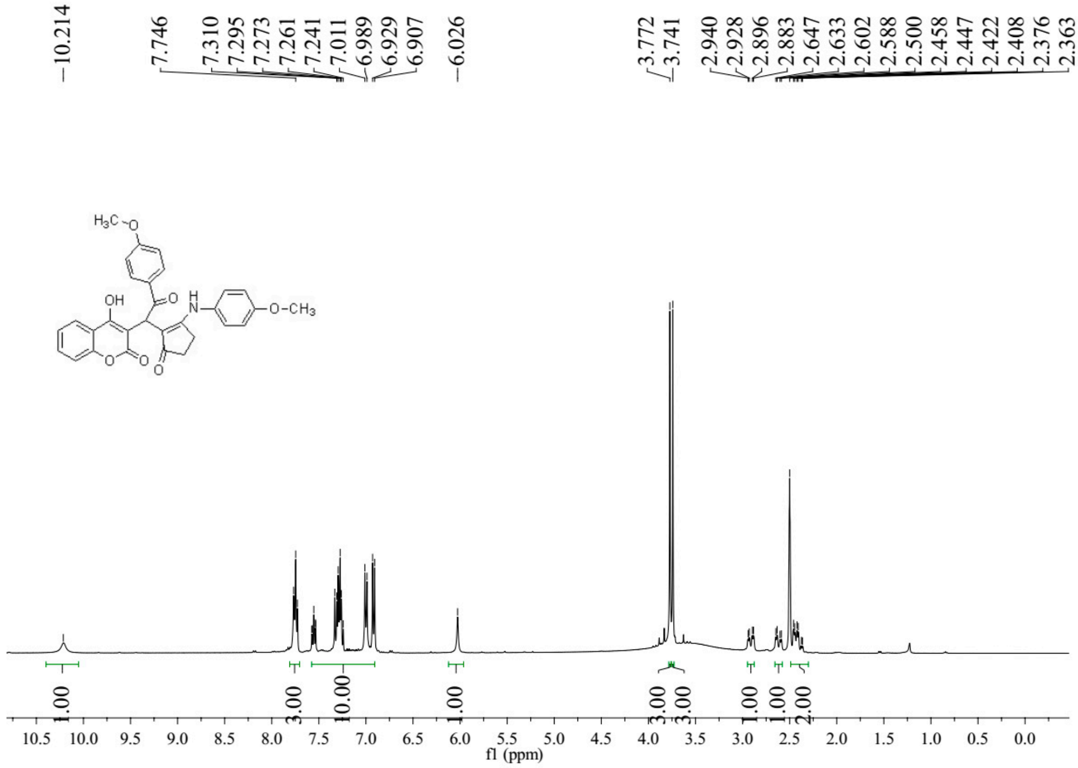

^{13}C NMR of compound **4f**

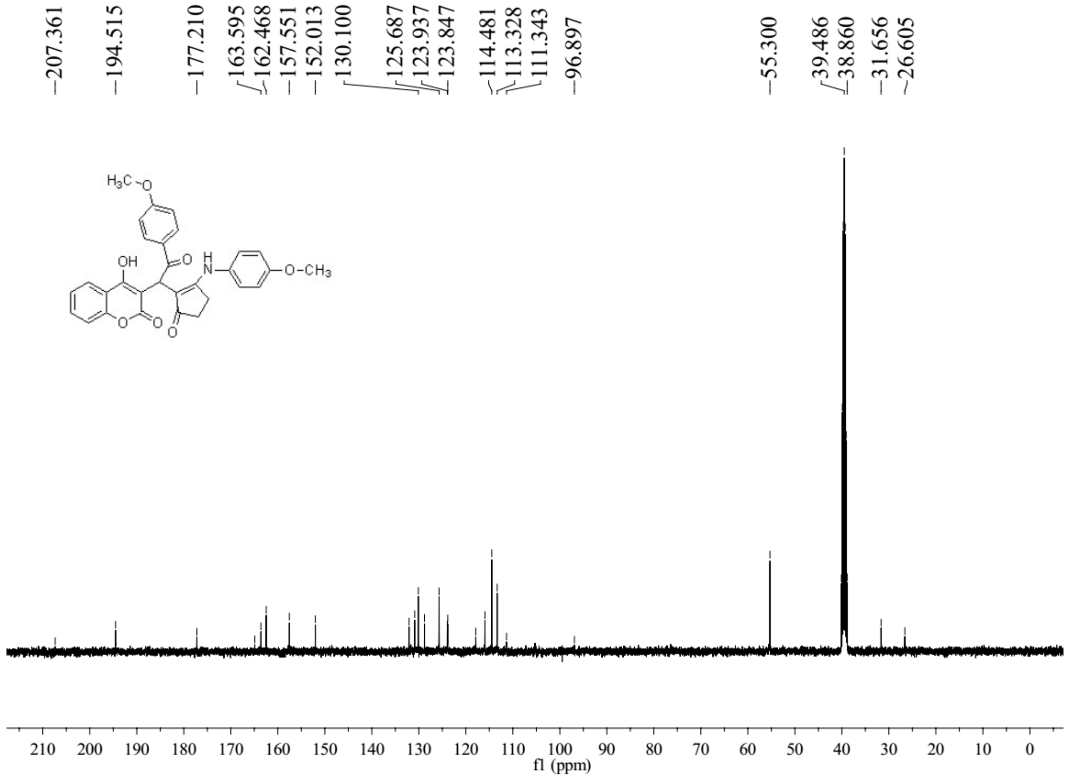

^{1}H NMR of compound **4g**

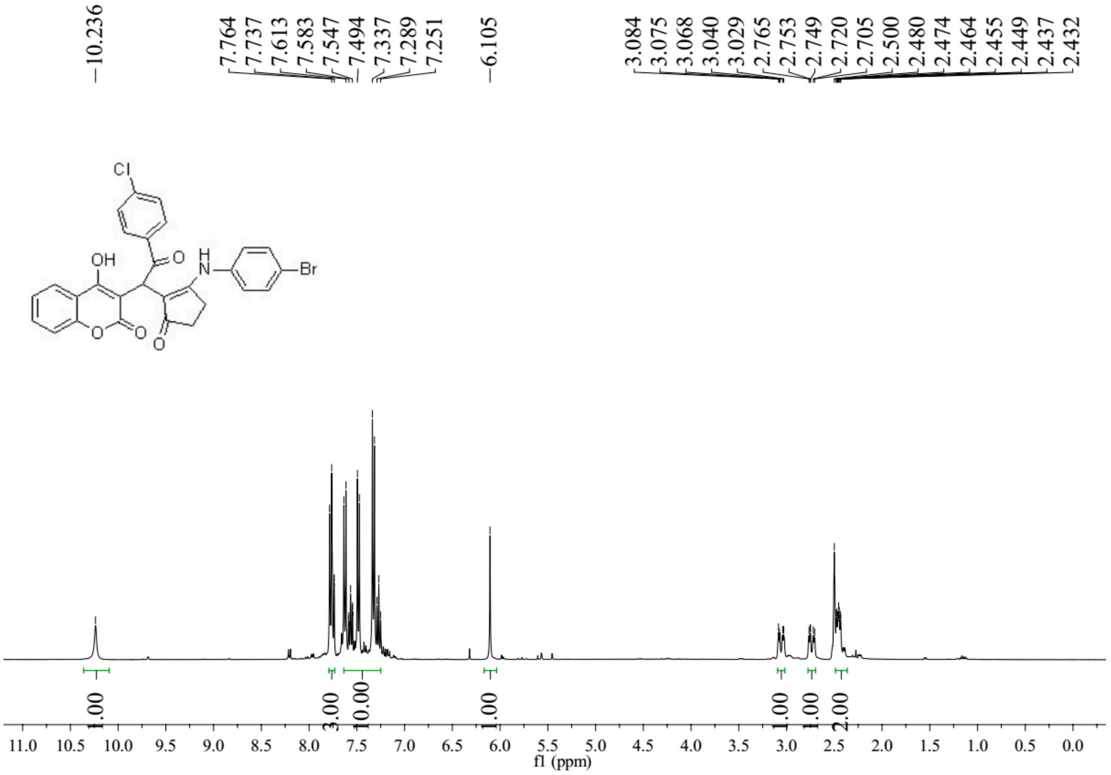

^{13}C NMR of compound **4g**

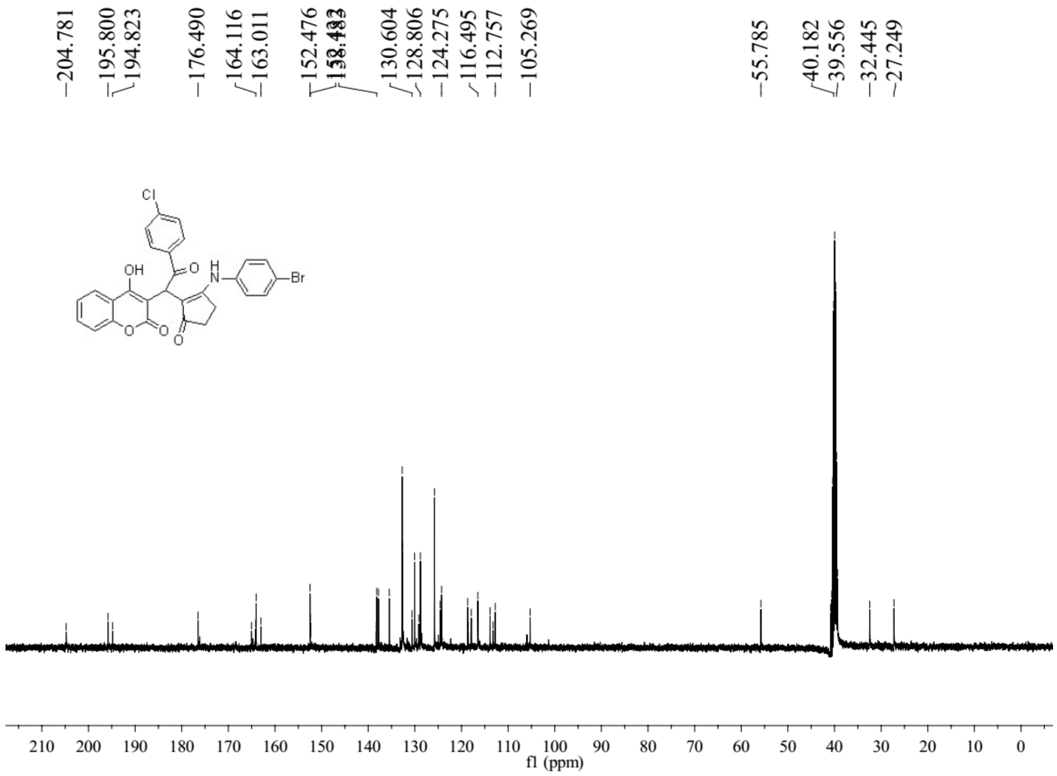

^{1}H NMR of compound **4h**

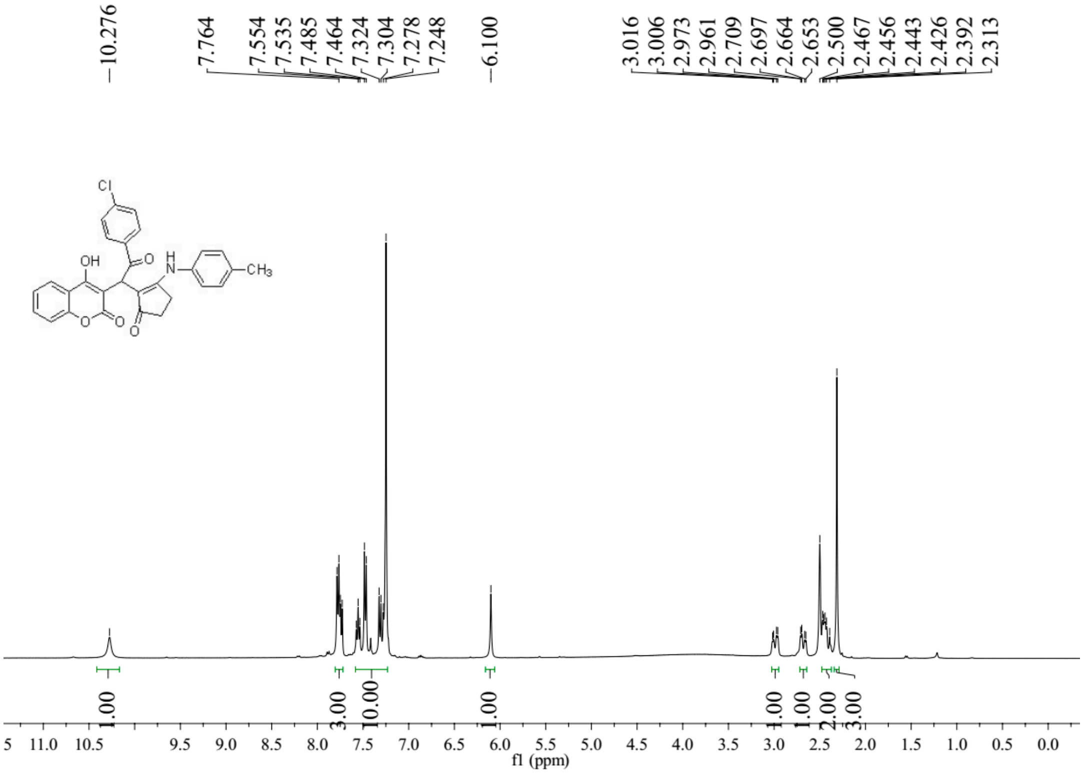

^{13}C NMR of compound **4h**

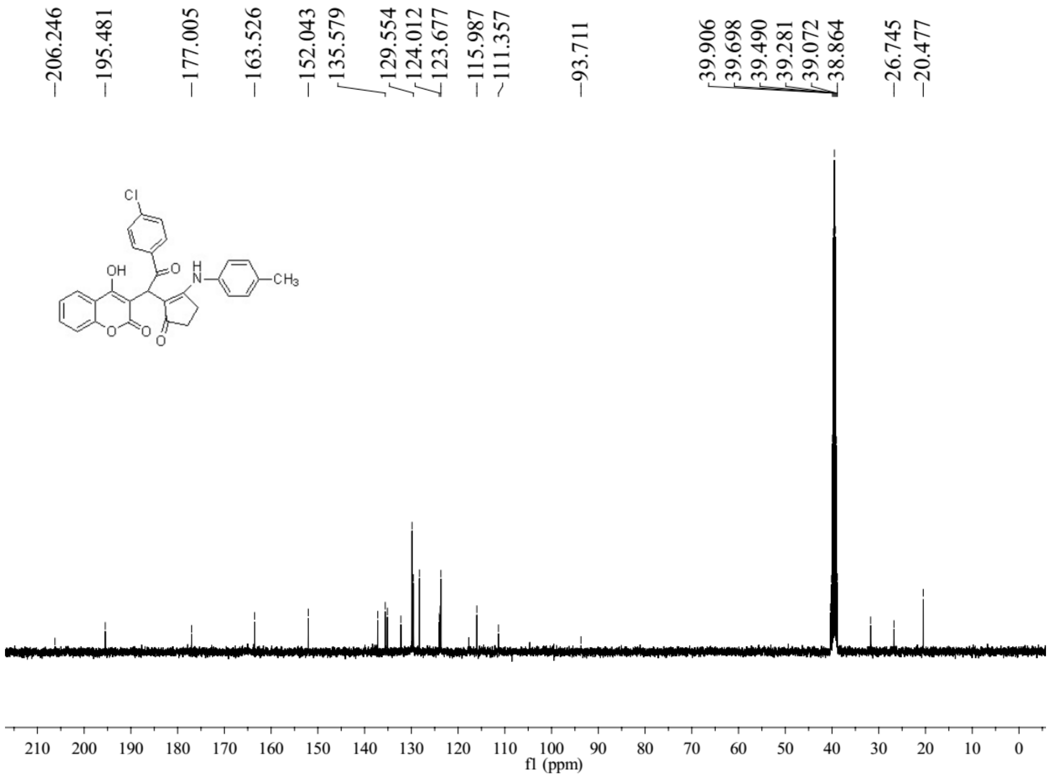

^{1}H NMR of compound **4i**

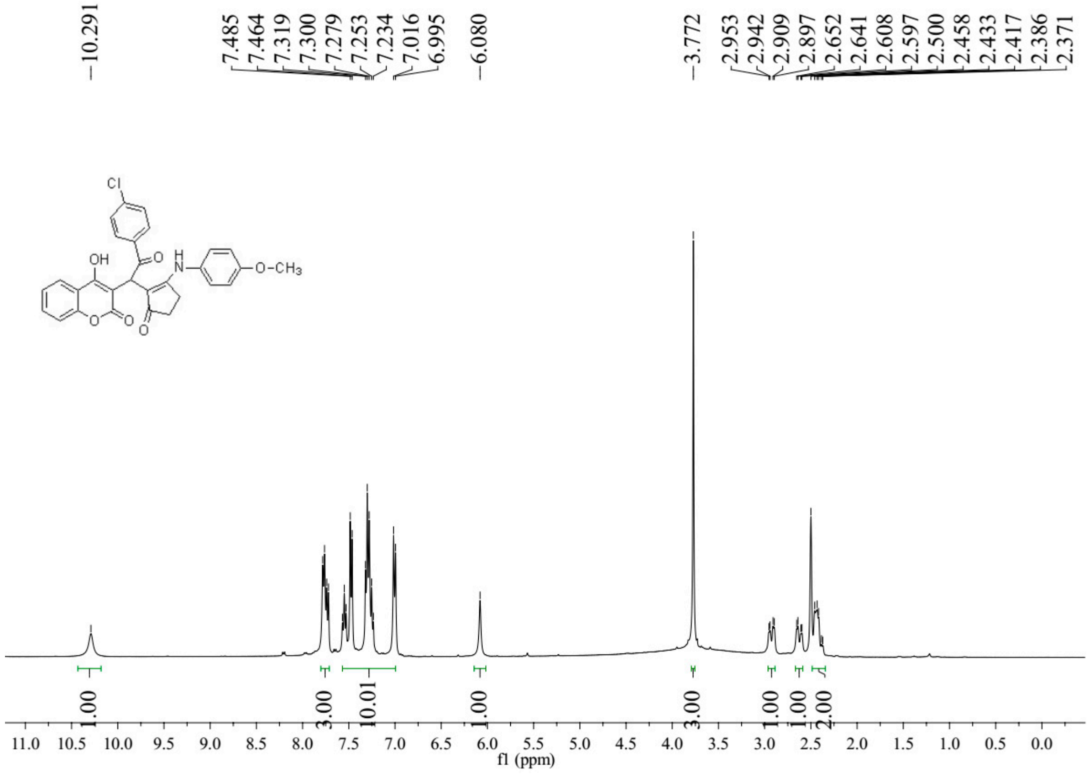

^{13}C NMR of compound **4i**

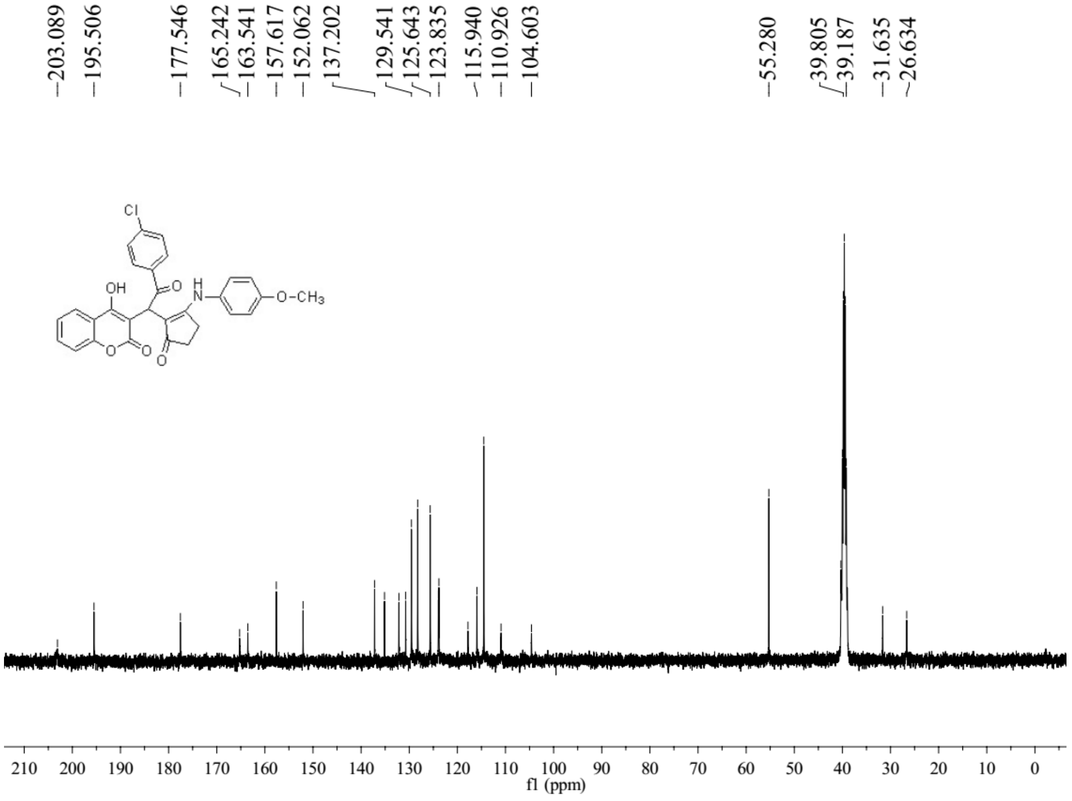

^{1}H NMR of compound **4j**

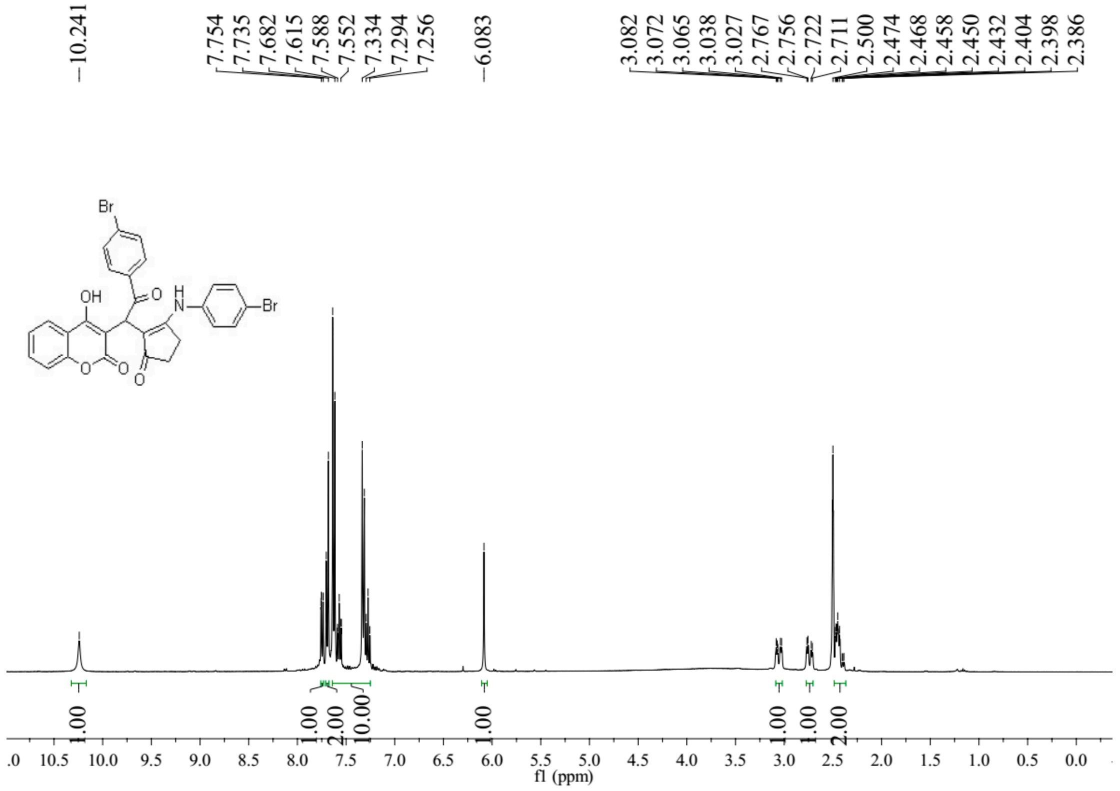

^{13}C NMR of compound **4j**

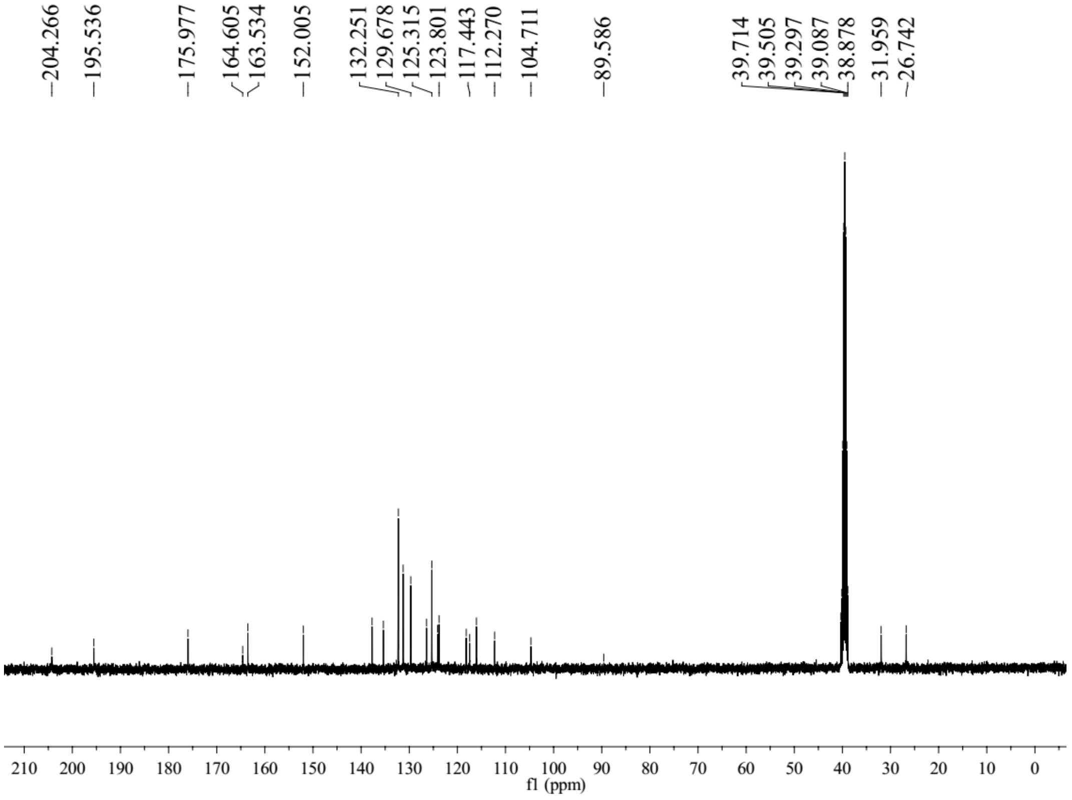

^{1}H NMR of compound **4k**

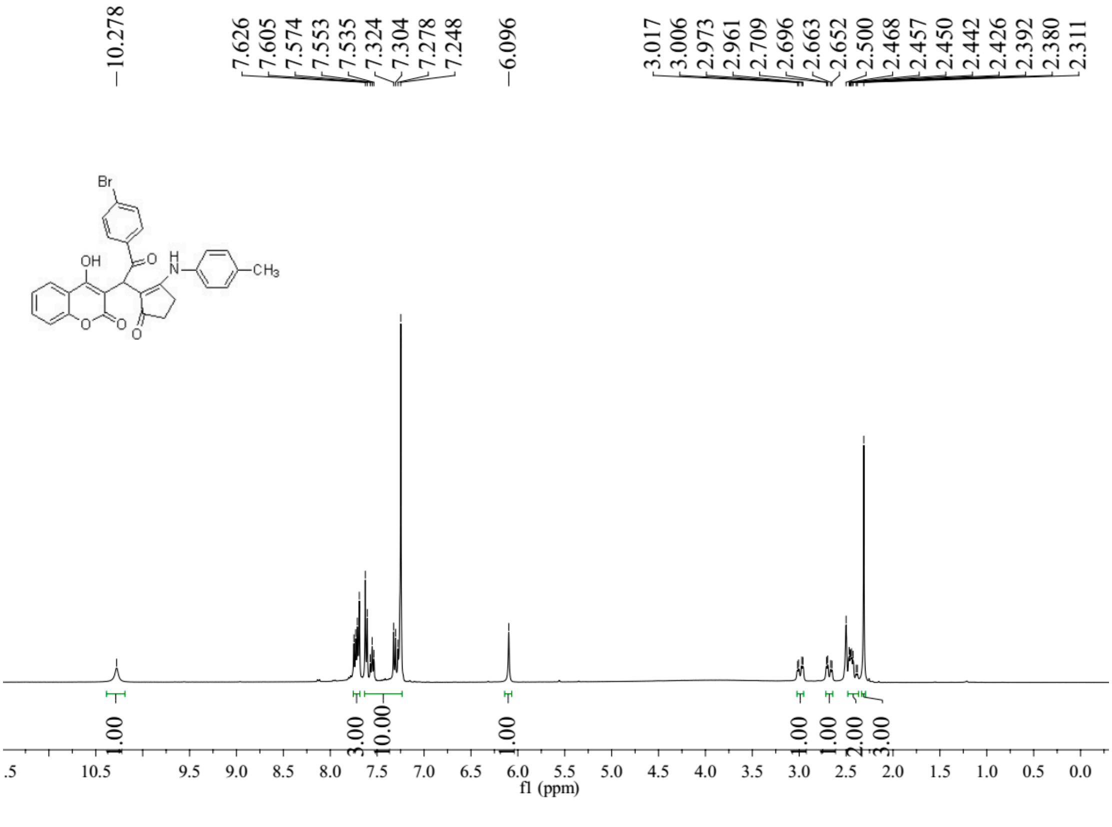

^{13}C NMR of compound **4k**

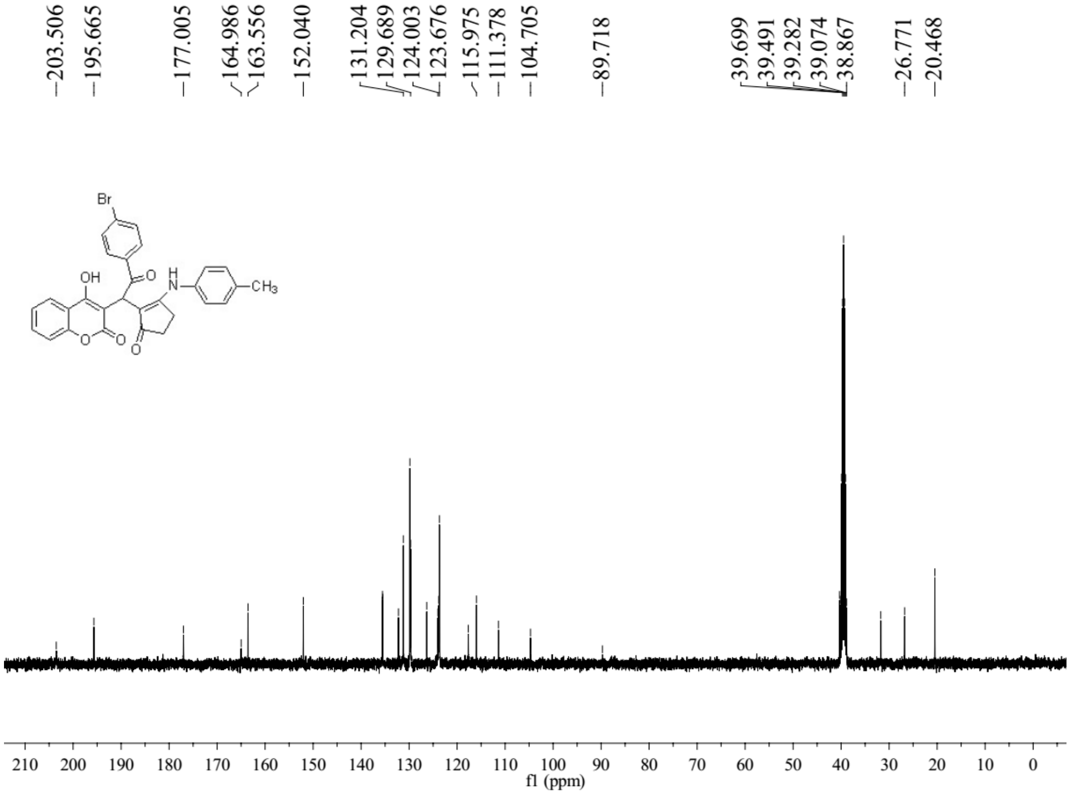

^{1}H NMR of compound **6a**

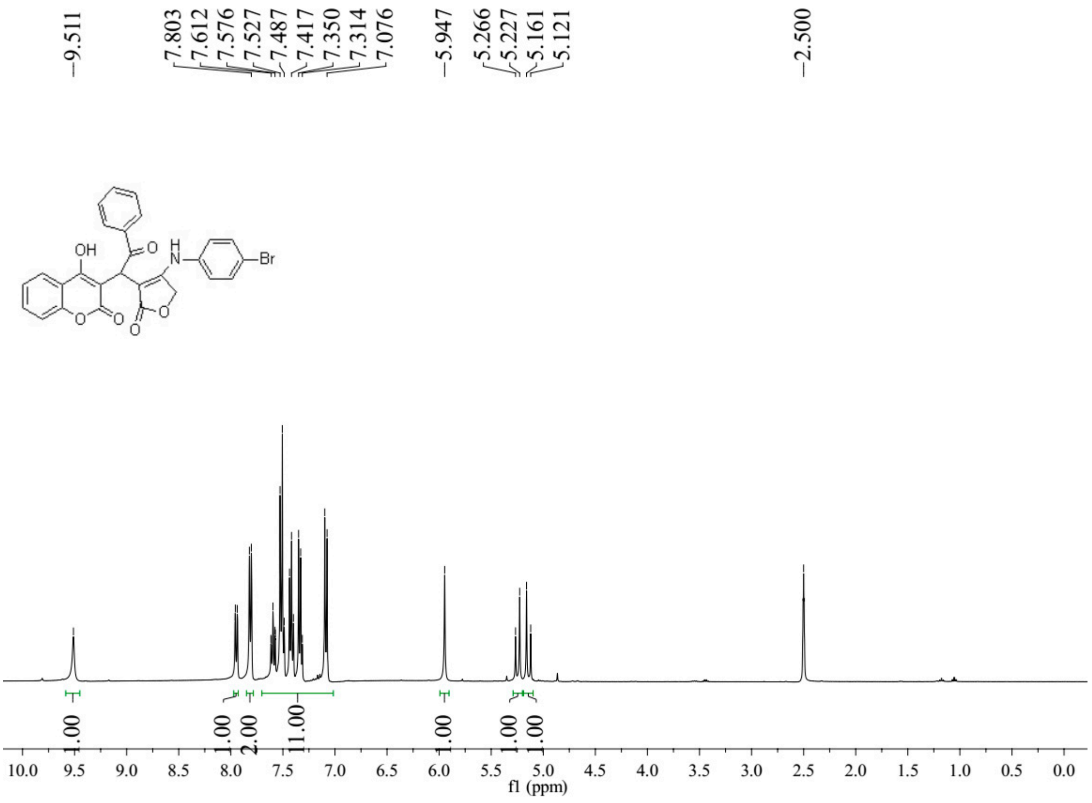

^{13}C NMR of compound **6a**

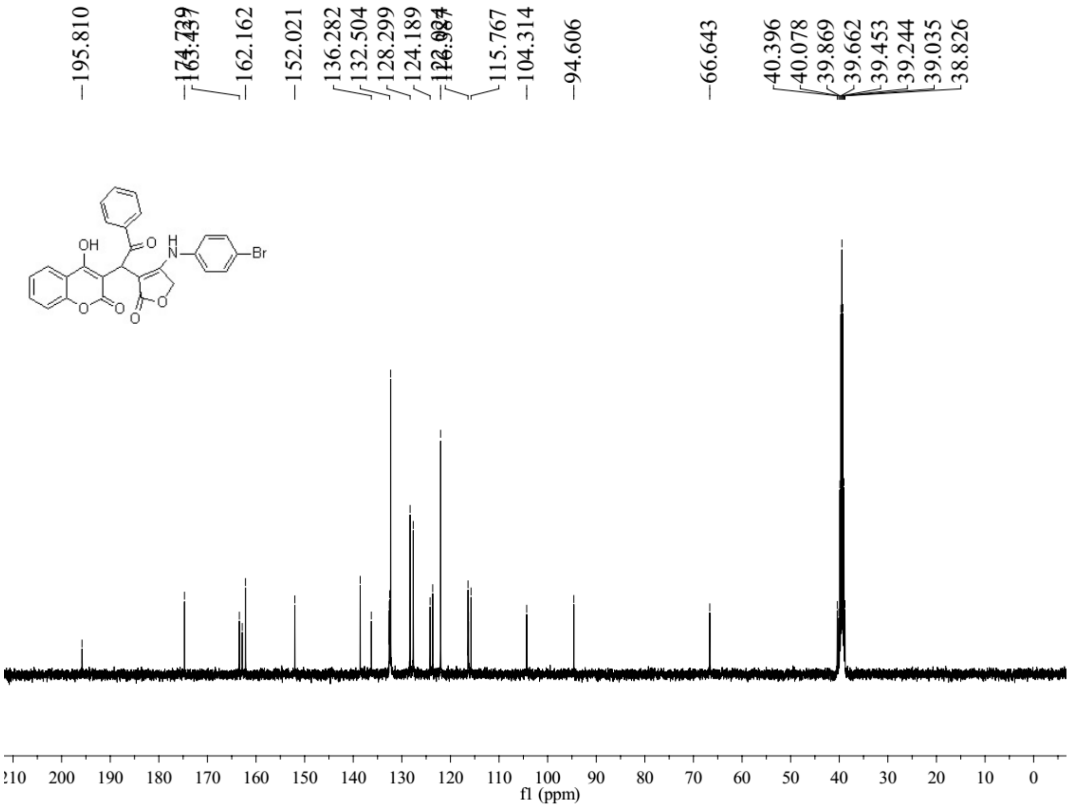

^{1}H NMR of compound **6b**

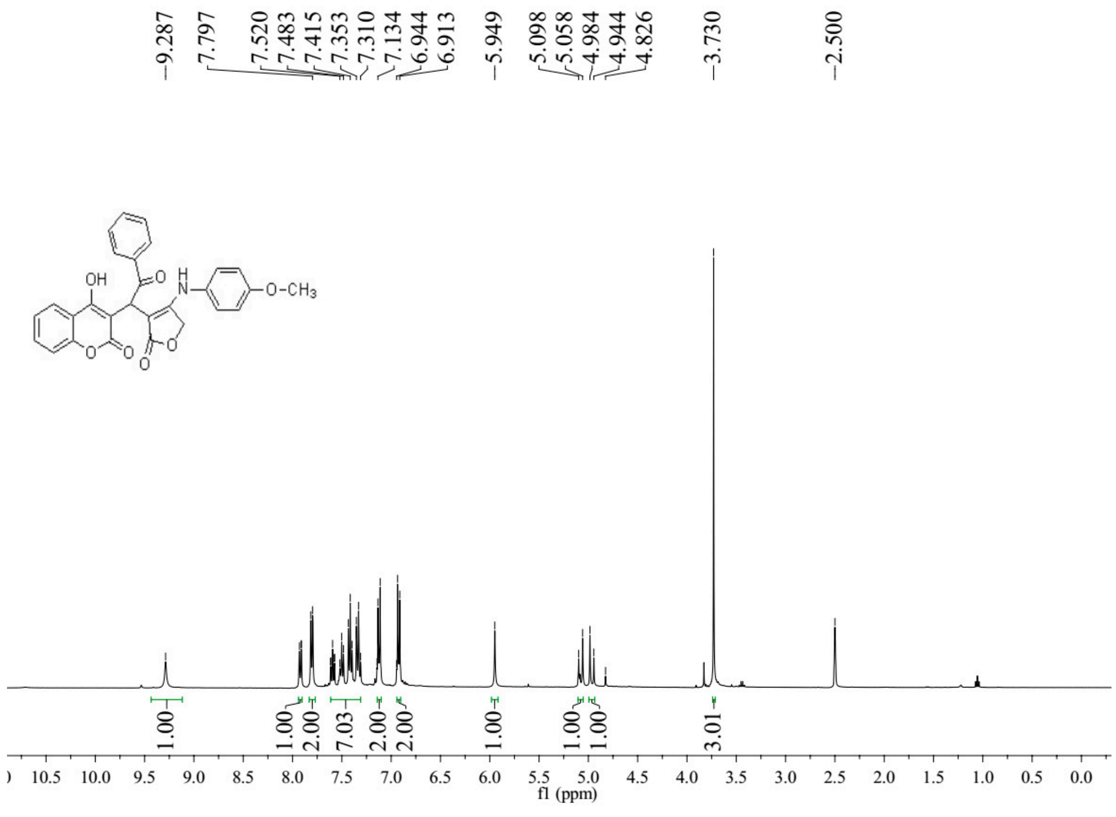

^{13}C NMR of compound **6b**

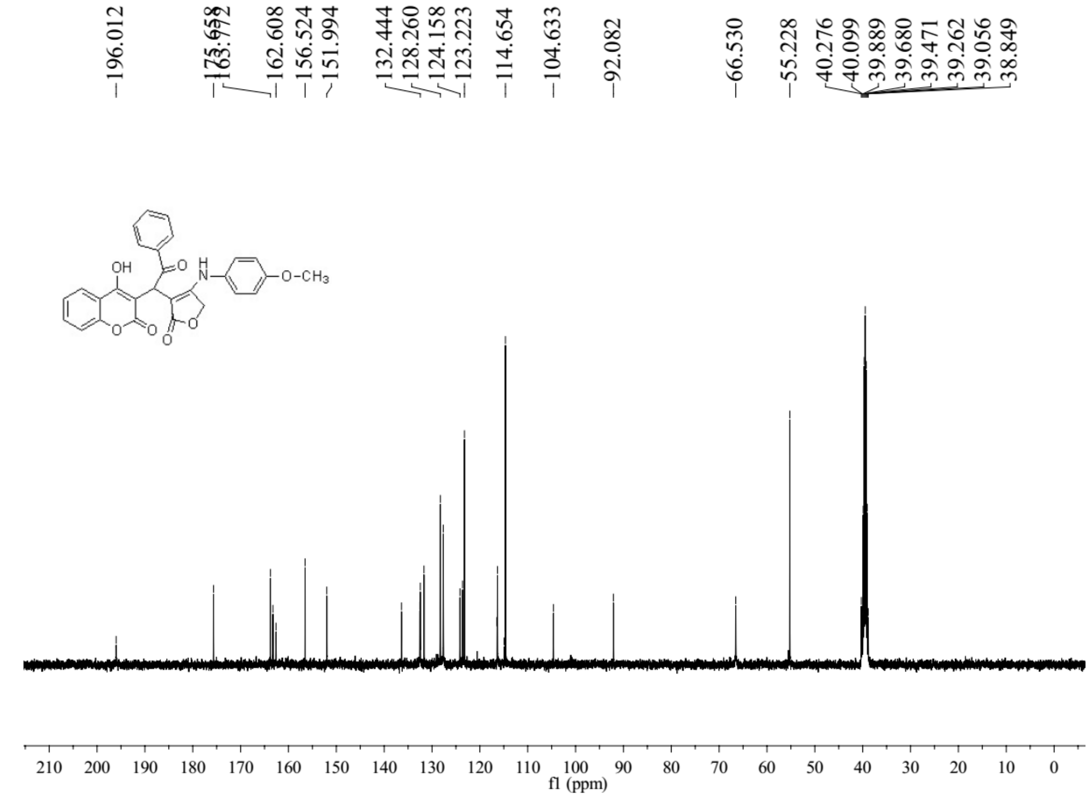

^{1}H NMR of compound **6c**

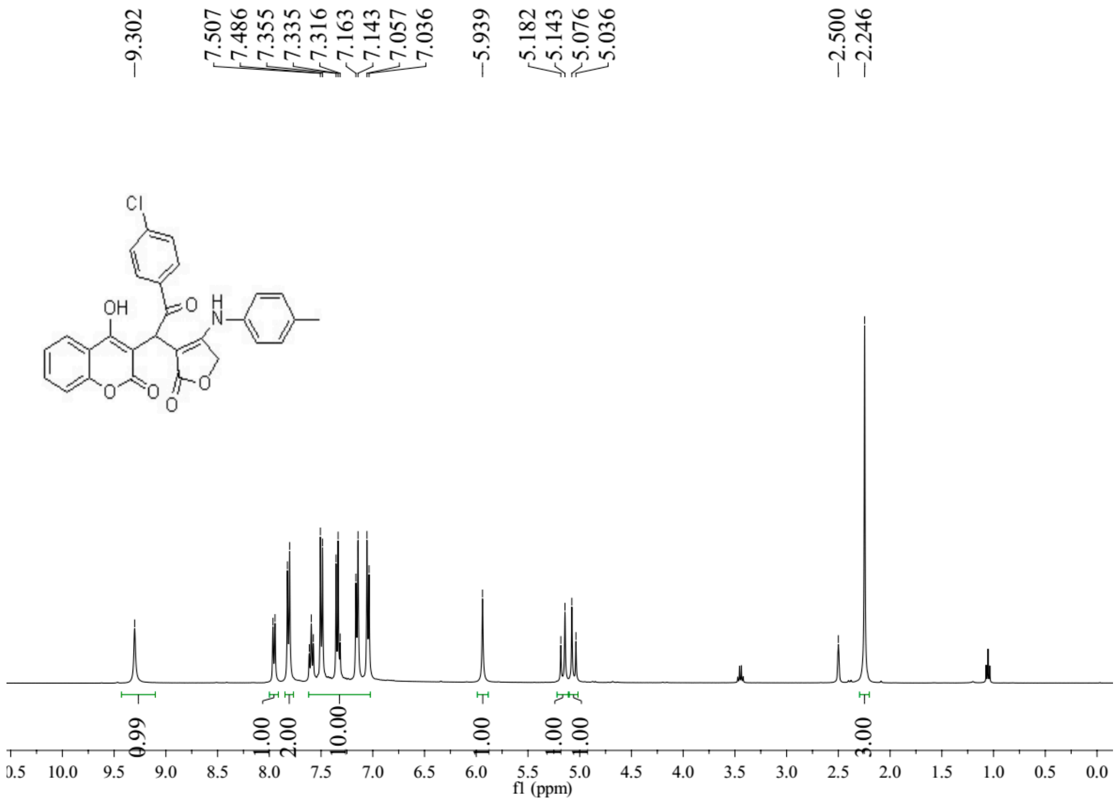

^{13}C NMR of compound **6c**

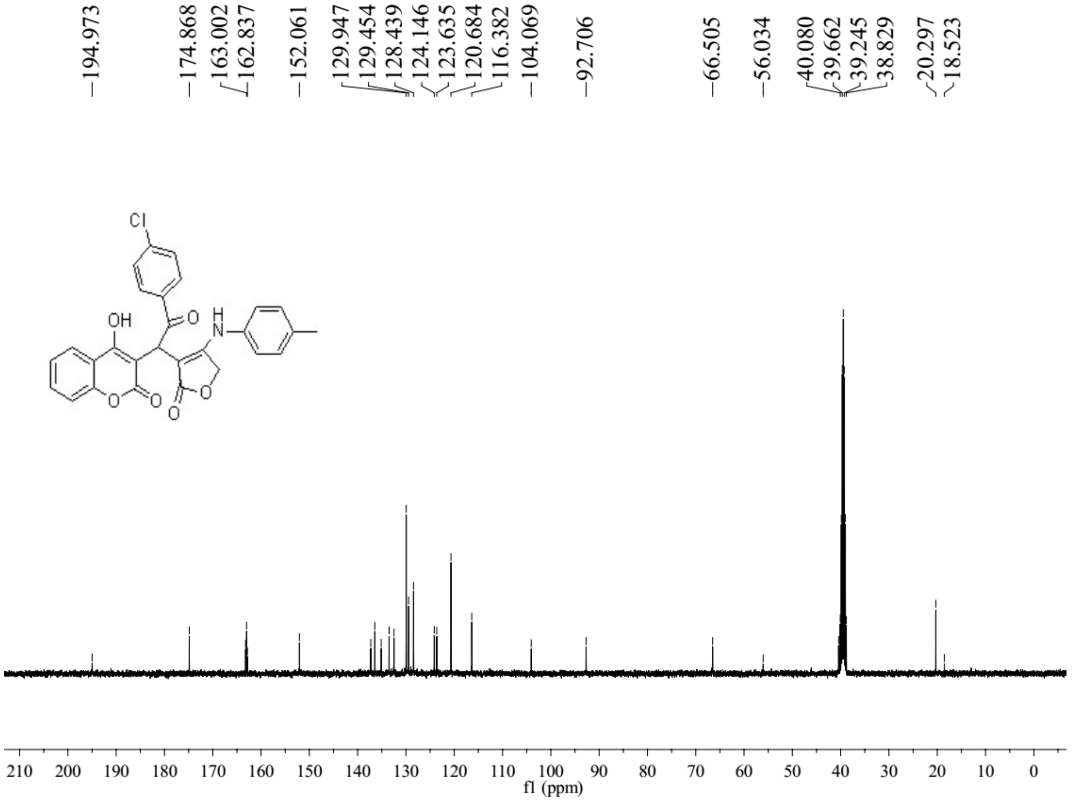

^{1}H NMR of compound **6d**

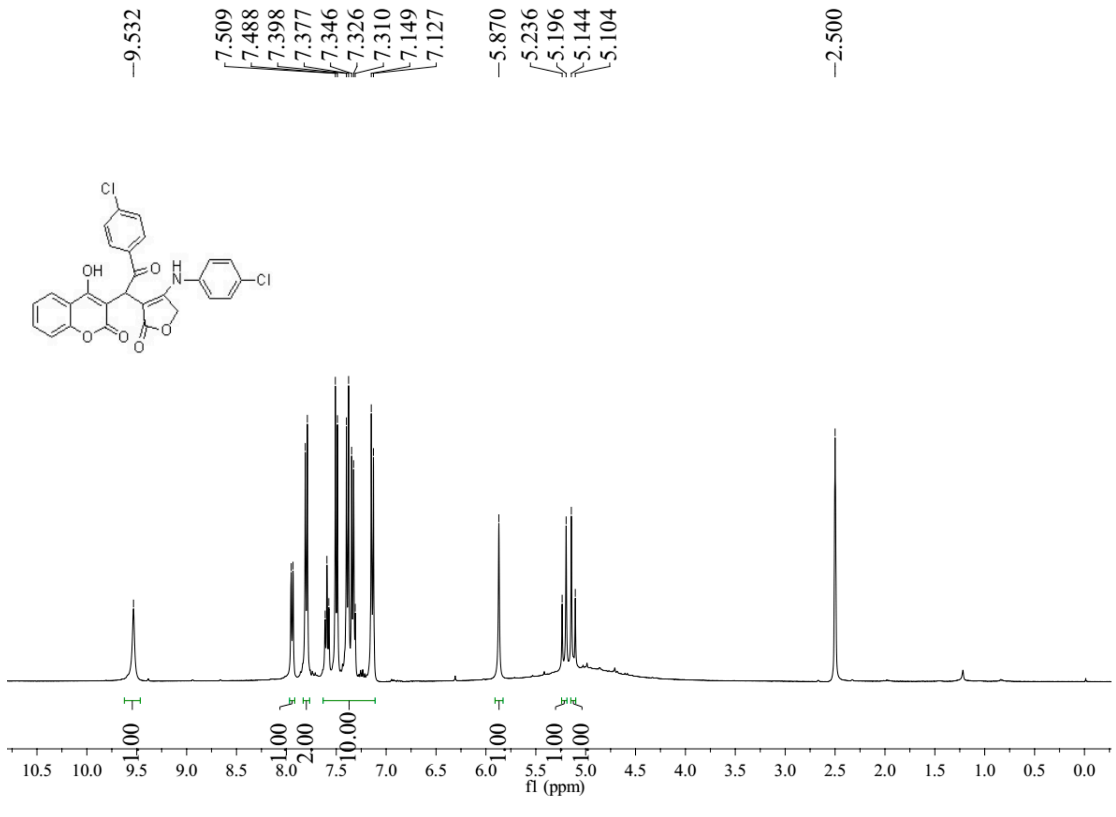

^{13}C NMR of compound **6d**

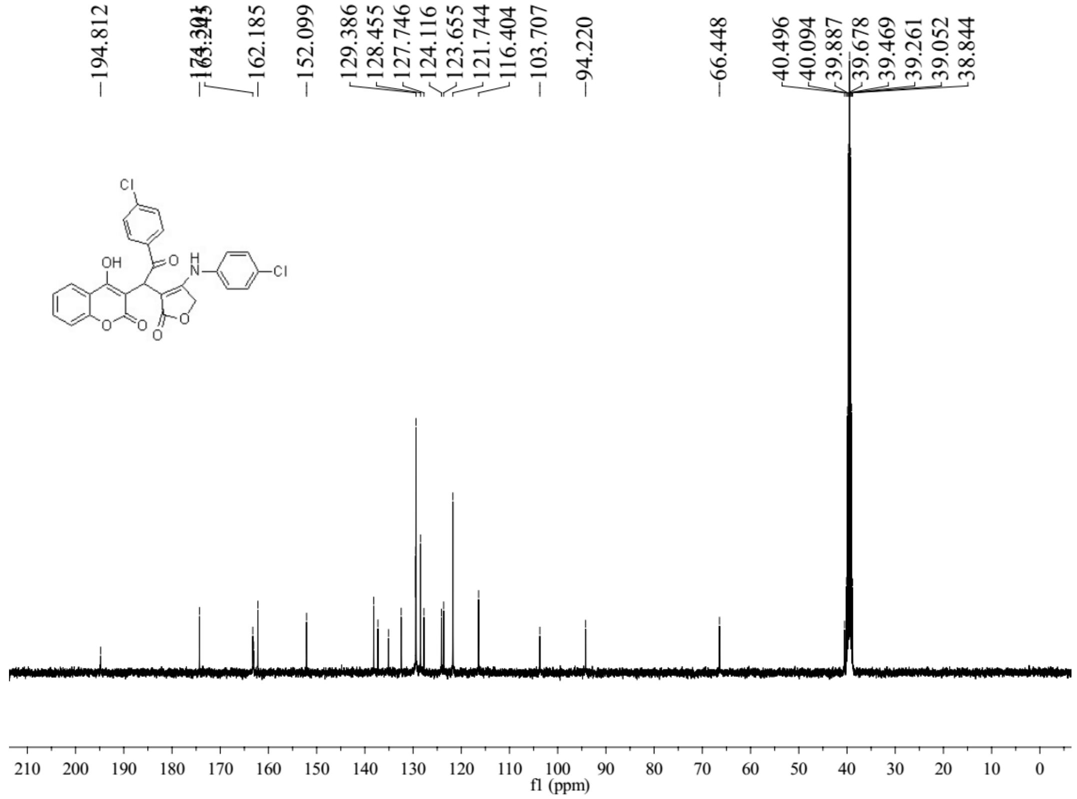

^{1}H NMR of compound **6e**

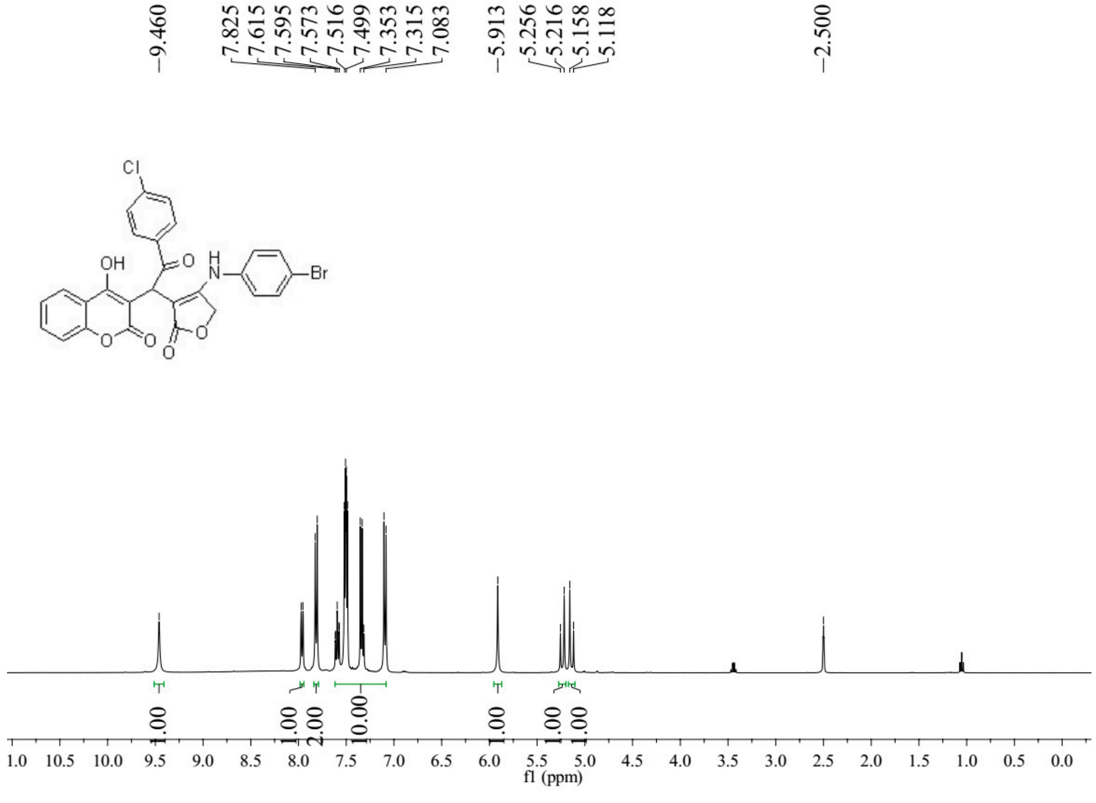

^{13}C NMR of compound **6e**

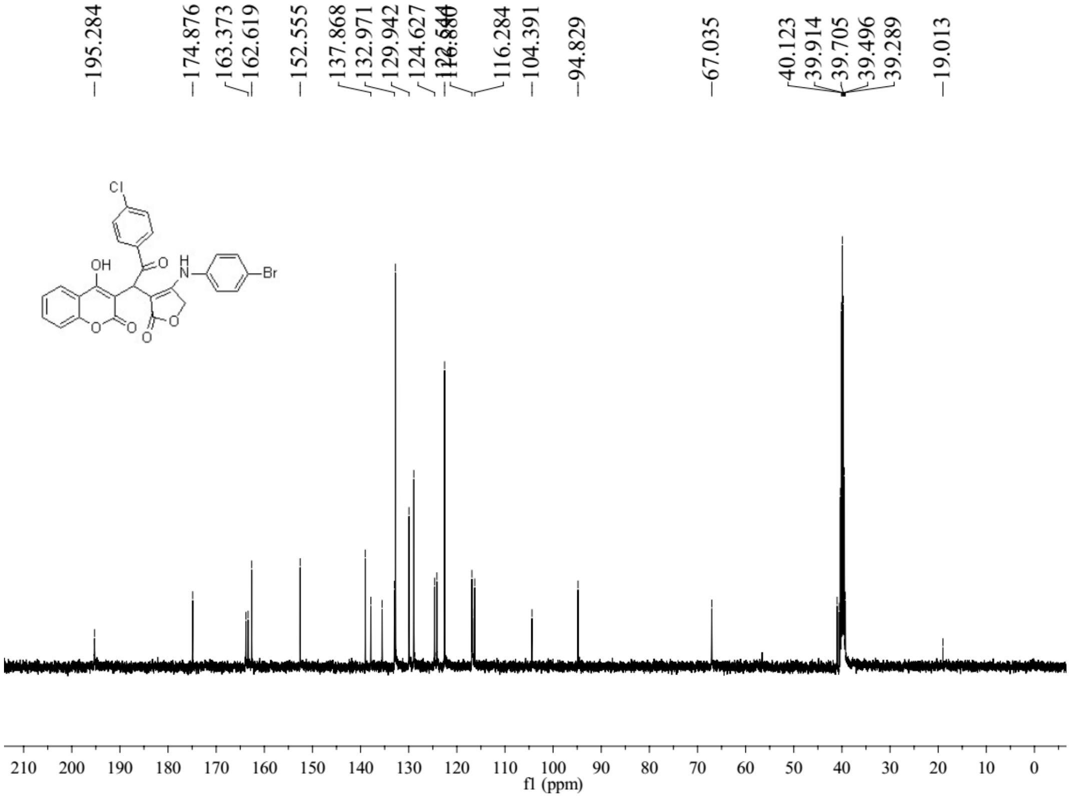

^{1}H NMR of compound **6f**

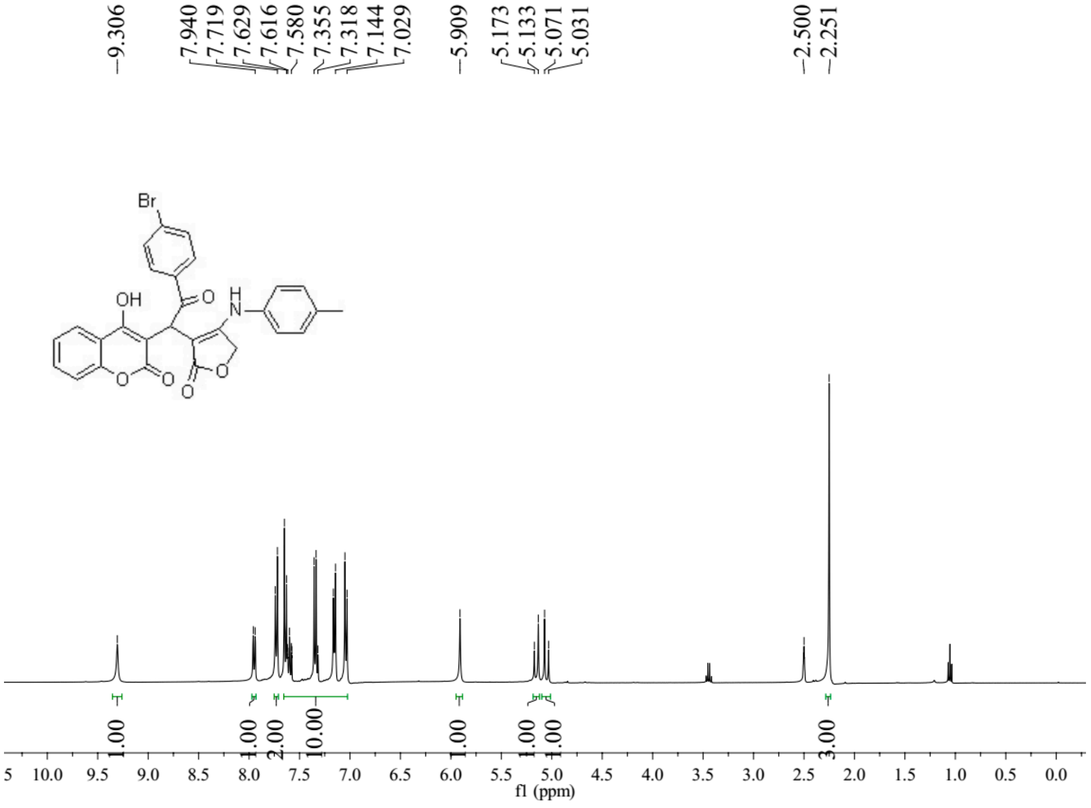

^{13}C NMR of compound **6f**

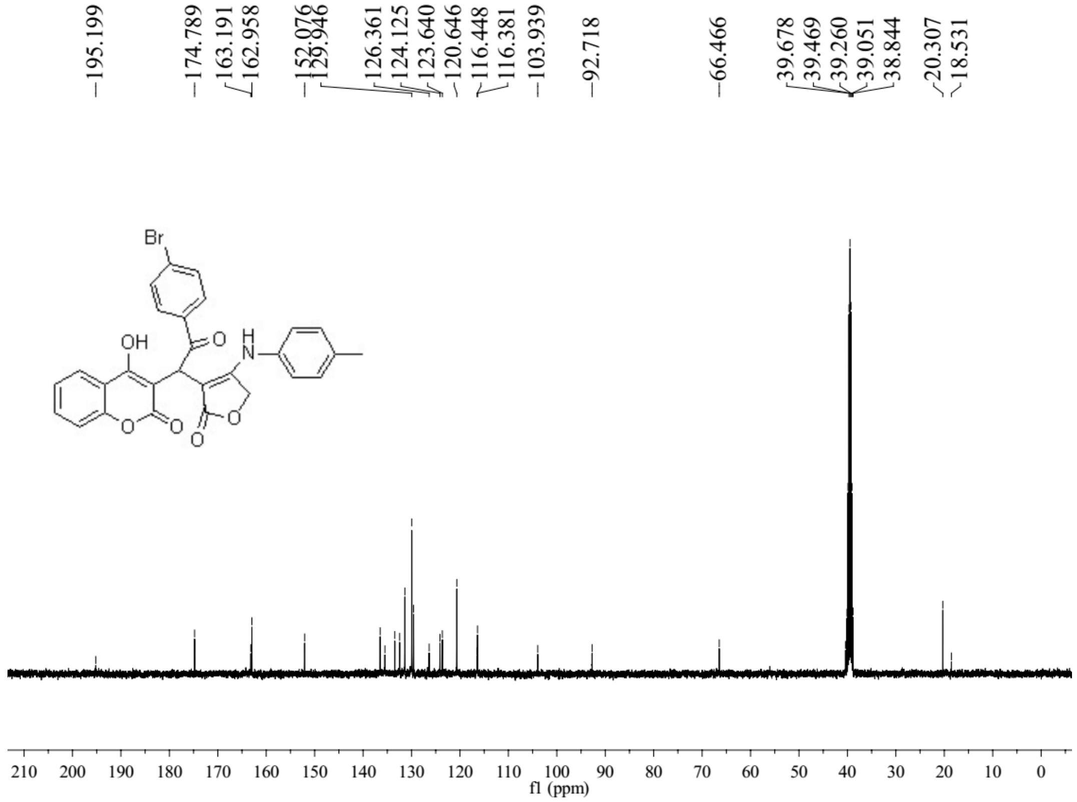

^{1}H NMR of compound **6g**

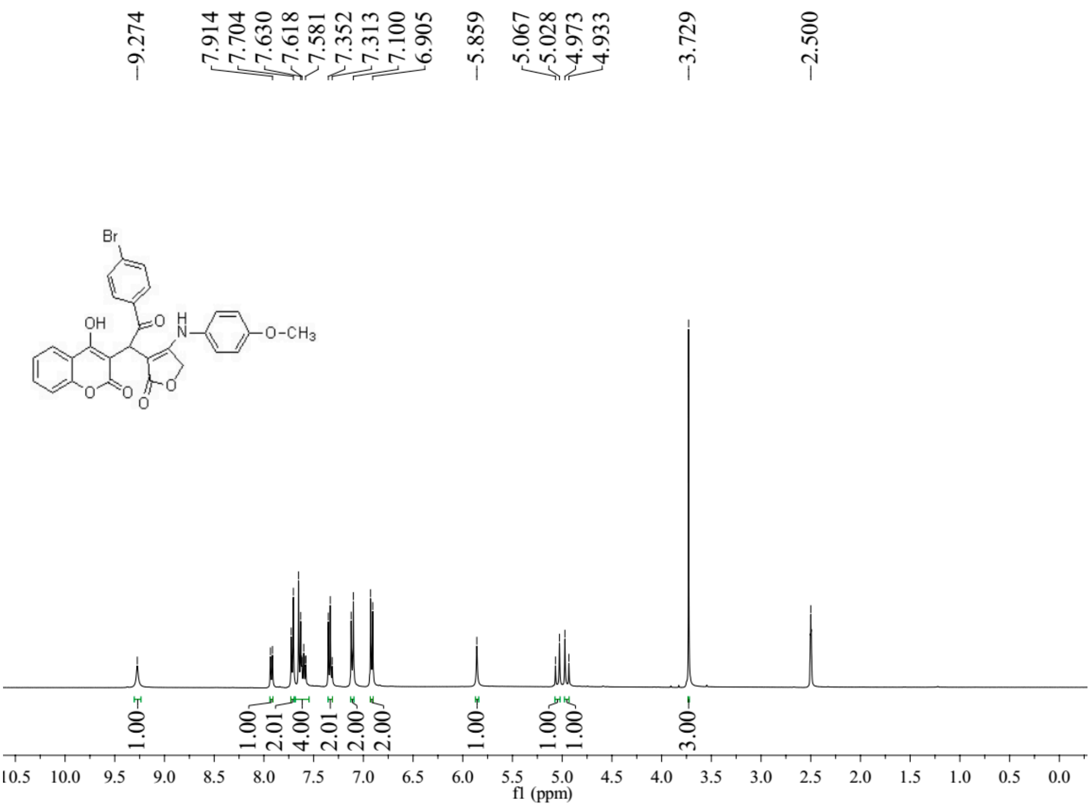

^{13}C NMR of compound **6g**

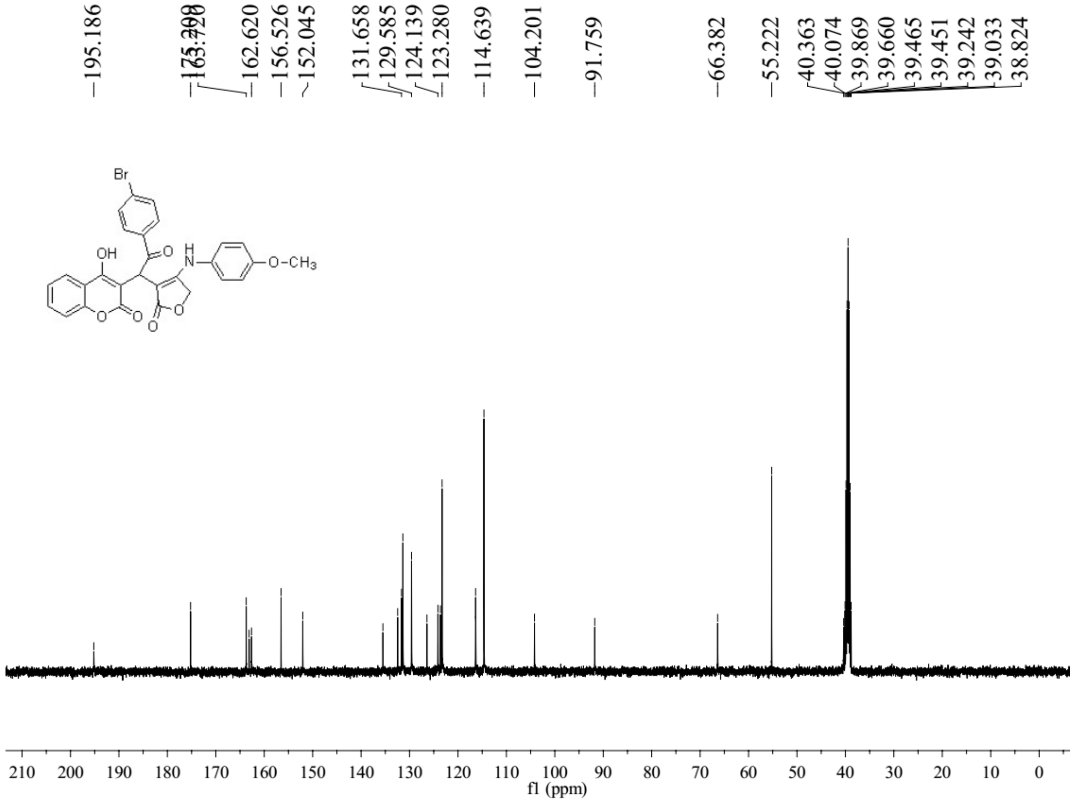

^{1}H NMR of compound **6h**

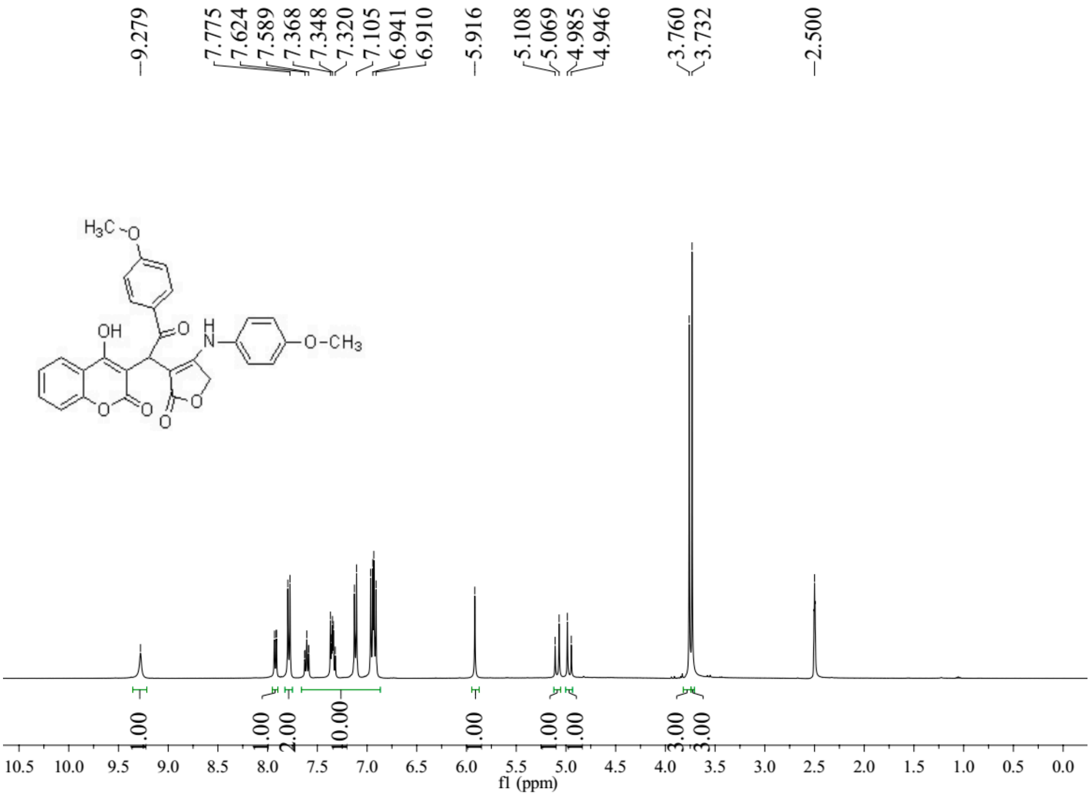

^{13}C NMR of compound **6h**

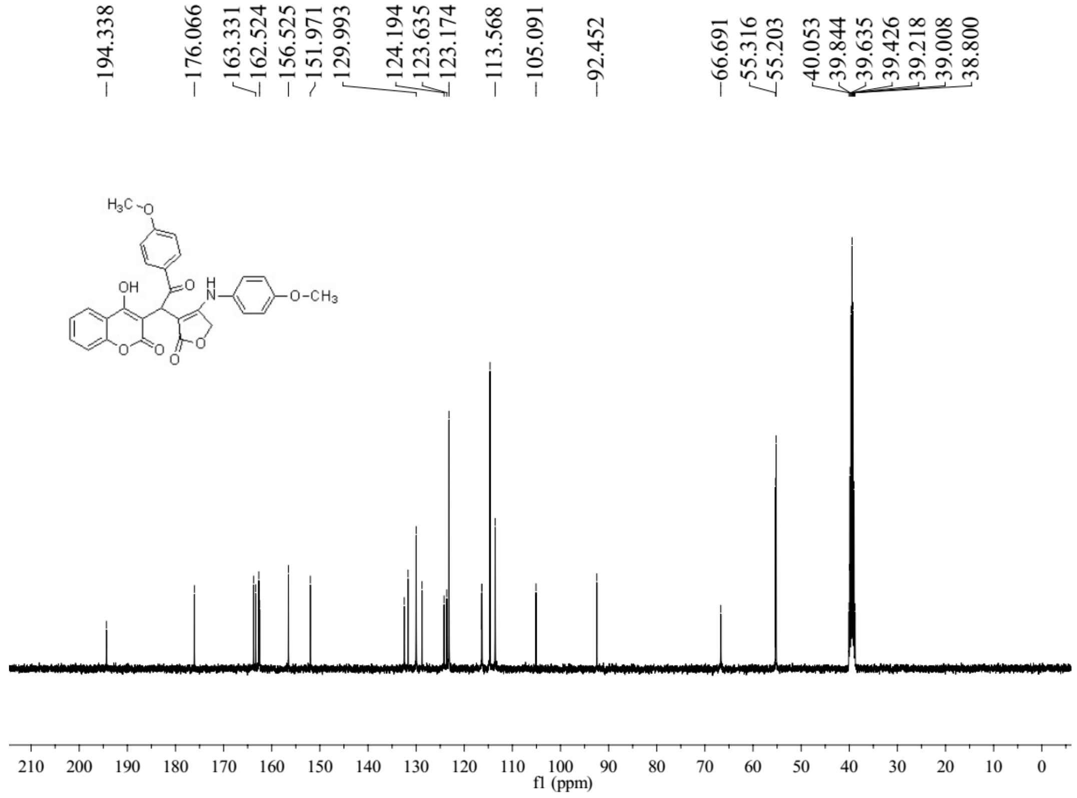

^{1}H NMR of compound **6i**

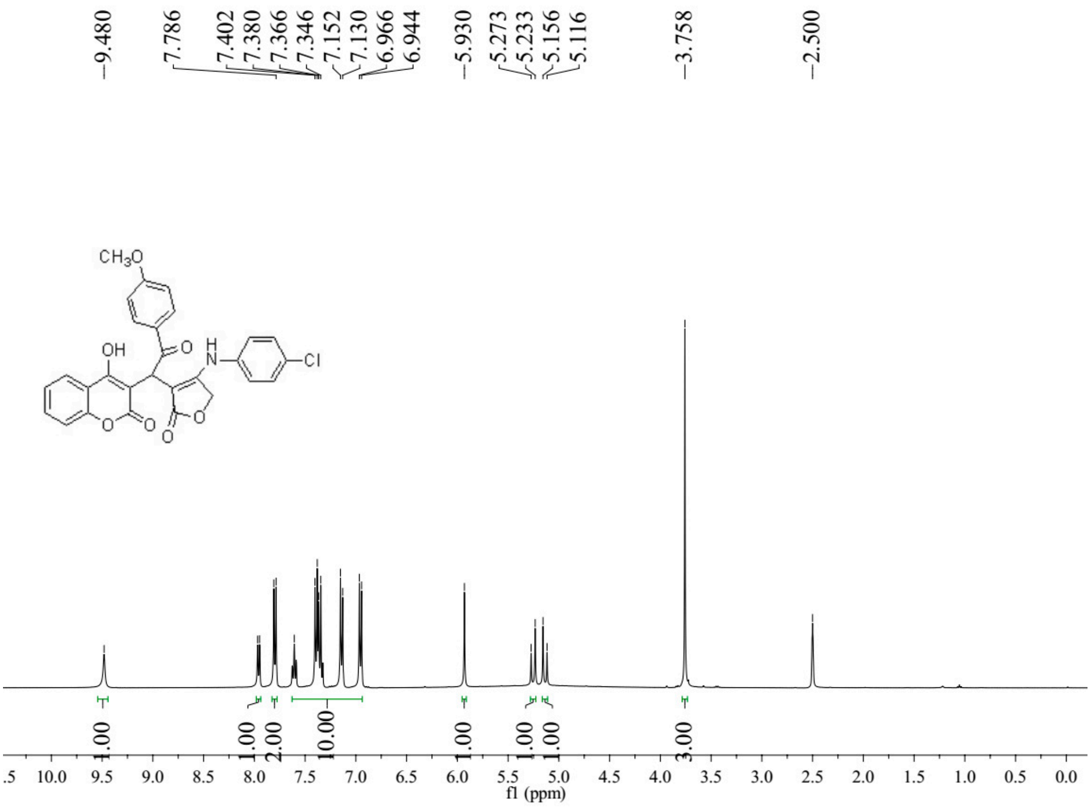

^{13}C NMR of compound **6i**

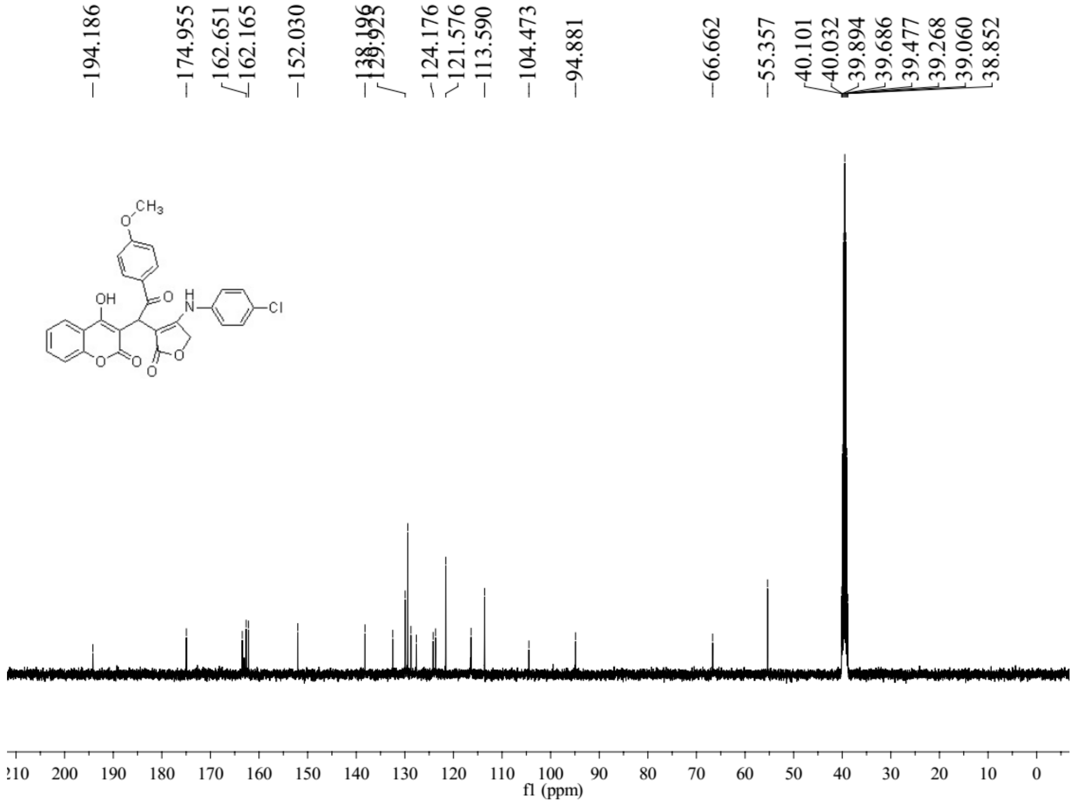

Supplement: Supplementary file 1 [file molecules-23-00235-s001.pdf]
